# Exploring Work Absences and Return to Work During Social Transition and Following Gender-Affirming Care, a Mixed-Methods Approach: ‘Bridging Support Actors Through Literacy’

**DOI:** 10.1007/s10926-023-10139-x

**Published:** 2023-10-21

**Authors:** Joy Van de Cauter, Dominique Van de Velde, Joz Motmans, Els Clays, Lutgart Braeckman

**Affiliations:** 1 Department of Public Health and Primary Care, Unit of Occupational and Insurance Medicine, Faculty of Medicine and Health Sciences, Ghent University, 10 Corneel Heymanslaan, 9000 Ghent, Belgium; 2 Department of Rehabilitation Sciences, Faculty of Medicine and Health Sciences, Ghent University, 10 Corneel Heymanslaan, 9000 Ghent, Belgium; 3 Center for Sexology and Gender, Ghent University Hospital, 10 Corneel Heymanslaan, 9000 Ghent, Belgium; 4 Department of Public Health and Primary Care, Unit of Epidemiology and Prevention, Faculty of Medicine and Health Sciences, Ghent University, 10 Corneel Heymanslaan, 9000 Ghent, Belgium

**Keywords:** Gender identity, Transgender people, Occupational health, Health literacy, Return to work

## Abstract

**Purpose:**

Research on return to work (RTW) following transition-related gender-affirming care (GAC) is lacking. We aim to study the RTW outcomes and experiences of transgender and gender diverse (TGD) people during social and medical transition to understand their needs better and provide tailored support.

**Methods:**

In this convergent mixed-methods study, the questionnaires of 125 employed TGD people, who took steps in transition (social and GAC), were analyzed for personal- and work characteristics, medical work absences, RTW, support at work, and health literacy. In-depth interviews were held with twenty TGD people to explore perceived facilitators and barriers to RTW.

**Results:**

One hundred and nine participants reported an average of 38 sick days after GAC. The majority (90.2%) resumed their job at the same employer. Although TGD workers felt supported, their health literacy (55.1%) was lower compared to the general population. The qualitative data analysis revealed four major themes: (1) the need and access to information; (2) having multidisciplinary TGD allies; (3) the influence of the occupational position; (4) the precarious balance between work, life, and GAC. Especially participants with a low health literacy level experienced RTW barriers by struggling: (1) to find and/or apply information; (2) to navigate (occupational) health and insurance services.

**Conclusion:**

Our research has shown that RTW for TGD individuals is a multifaceted process, affected by personal factors, work-related elements, and the characteristics of the healthcare and social insurance system.

Enhancing support for TGD people at work and their RTW requires a high need for centralized information and promoting health literacy while engaging relevant stakeholders, such as prevention services and employers.

**Supplementary Information:**

The online version contains supplementary material available at 10.1007/s10926-023-10139-x.

## Introduction

### Gender Identity, - Transition and - Affirming (Health) Care

Worldwide, the transgender and gender diverse (TGD) population is ever more growing, with estimated proportions of 0.02 to 0.08% in adults among health system-based studies and 0.3 to 0.5% by way of self-report [1] (for definitions see ‘terms of reference’ in Online resource 1). In the last national survey [2] mapping the landscape and experiences of TGD people in Belgium (2017), almost half (47.9%) of 534 respondents self-identified as transgender women (TW), while 27.9% identified as transgender men (TM) and 23.2% as gender diverse (GD) people and 3.7% indicated cross-dressing as their gender identity/expression.

Separately or associated with a social transition (changing name, pronouns, clothing, hair, body language), TGD people can opt for gender-affirming medical and surgical interventions to align their body with their gender identities, such as hormonal therapy, surgery, and additional supporting treatments comprising psychosocial counseling, electrolysis, speech therapy. Hormonal therapy for feminine embodiment goals consists of estrogen and androgen lowering medication, whereas for masculinization goals, this consists of testosterone [1, 3]. Gender-affirming surgery (GAS) consists of multiple possible procedures and is categorized according to assigned sex at birth (male versus female) [1). Most frequently reported studies [1] include genital-urological procedures (vaginoplasty, phalloplasty, metaoidioplasty), top surgery (mastectomy, breast augmentation), and facial surgery. TGD people opt for these treatments in an individualized way [1] based on personal wishes, financial resources, and the extent and severity of the procedure. The wish for gender-affirming medical and surgical interventions (GAMI) is more reported for TW and TM [1], such as in the recent ‘European Network for the Investigation of Gender Incongruence’ (ENIGI) study [4], where hormonal therapy and vaginoplasty were opted the most by TW and masculinizing hormones and mastectomy were the most frequent treatment in TM. It still remains unclear what proportion of GD individuals choose hormonal and/or surgical interventions to none at all as they also encounter higher rates of barriers to care [1, 5–8].

### (Return to) Work

Socio-medical transition while being employed is also often a stressful and a precarious socio-economic period as TGD people often face difficulties in accessing employment [2, 9, 10] and sustainable work [2, 11, 12]. For the latter, this is partially due because they face stigma and discrimination [13–16]; GD people even experience an exhausting amount of microaggressions [17] and may as such experience more stigma compared to TM and TW, as also noted by Dray et al. [18] in their study on TGD people’s general work experiences. Earlier work [19] found that TW had the worst work experiences, followed by GD individuals. Meanwhile, a recent study in the UK [20] found that TM who had less consistent masculinity traits were penalized at work. However, a national survey in Belgium [2] showed no significant differences in general work experiences between gender identity groups. These experiences of stigma and their anticipation leads to minority stress [21, 22] which is an unique added stressor making the TGD population more vulnerable to poor physical and psychological health outcomes [1] Furthermore, the socio-medical journey can result in frequent short and some long-term work absence(s), the latter needing a more formalized RTW process, often with assessment and support from medical professionals. For some TGD employees, this has had major implications, as absenteeism was the official reason for discharge [11].

The general focus of RTW research has mostly lain on a binary gender approach with mixed RTW results for men versus women [23–25]. Earlier RTW research and reviews on a variety of common psychosocial and medical conditions have identified several factors linked to employment and job resumption after (sickness) absences [23, 26–31] which relate to the dominant biopsychosocial model approach[32]: (1) personal characteristics (e.g., age, education, expectations, coping, interpersonal problems [33], health literacy [34, 35]; (2) medical characteristics (e.g., disease severity, comorbidities); and (3) work- related characteristics (e.g., job demands, social support [36]. All these factors can be seen as either a facilitator or a barrier to returning to work. Notwithstanding the views on gender as a social construct, the issue of employment, sick leave, and work resumption of TGD people has only recently been approached and reported [37]. This recent systematic review [37] showed a gap of knowledge regarding objective data on work absences (sick days) and return to work following GAMI. According to one study from 2010 [11], which had 610 TGD participants, the average yearly general absenteeism of TGD individuals who did not wish to undergo GAS was two weeks compared to 5 weeks for those who had GAS, but this absenteeism was attributed to psychosocial factors. In addition, no readily available data exist on specific job-re-entry experiences of TGD people, possible work accommodations, and the role of occupational health professionals [37].

### Health Literacy and (Return to) Work Support

As indicated by Levesque [38] and emphasized by the ENIGI study [4], access to healthcare and navigating support systems at work may on an individual level be influenced by (health) literacy, socioeconomic status, and identity factors. Health literacy (HL) is a dynamic fluctuating concept within a person’s life and is a shared responsibility of the individual, society, and healthcare systems [39, 40] and crucial for a supporting work environment [41]. Several models and definitions exist for the concept of HL such as the behavioral- (e.g. self-control, communication, etc.) and perceptions- (self-perception, being proactive towards health) focused model by Soellner et al. (2017) [42]. Another HL model was created by Sørensen et al. (2012) [43] on which the European Health Literacy Survey (EU-HLS) was built (see ‘Online Resource 1’ for more information on both HL models). The last Belgian national study [44] using the short form of the EU-HLS [45] showed that 33.4% possessed limited HL, however, gender-specific results were only reported in a binary manner.

While support, a social determinant of health and essential RTW factor [28, 36], is often reported in TGD health studies [46–48] as a mediator for work experiences [4, 13, 49] and plays a role in accessing care [50, 51], the emphasis is placed upon support received from coworkers, supervisors, family, and friends. The possible contributions of occupational health professionals are seldom mentioned in these TGD (health) studies, and health literacy evaluations are scarce. Moreover, although HL research recommends capacity building of the individual and the work environment in favor of a smooth RTW process, there are few studies of this combined focus [34, 35]. Therefore, mapping health literacy within the support systems of TGD people’s (return to) work experiences can contribute to multidisciplinary gender-affirming care.

### Study Aims and Objectives

A better understanding of work absences and returning to work during transition and after gender-related affirming care is important as strategies to facilitate RTW in TGD people could prevent different related personal, social, and economic problems. Given the scarcity of research on this topic, mixed methods research will allow for RTW exploration at a detailed level for a more nuanced understanding beyond the capabilities of either quantitative or qualitative approaches alone. Therefore, this exploratory study aimed to: (1) measure the work absence and RTW rate of TGD people after steps in gender transition; (2) explore their RTW experiences to add context; (3) explore general support at work during socio(medical) transition; (4) measure the health literacy level; and (5) explore the perceptions of TGD people on the role of the occupational physician and occupational health services.

## Methods and Design

### Setting and Context

In Belgium, according to legislation [52], each employer is obliged to be affiliated with an occupational health service (OHS), and every salaried worker has free access to an occupational physician (OP). OHS are services entrusted with preventive functions and are responsible for advising the employer, the workers, and their representatives to establish and maintain a safe and healthy work environment. OPs can assess the physical and psychosocial risks in different work environments, work with employers and other healthcare professions to develop strategies to address these risks effectively, and prove valuable to more vulnerable workers [53, 54].

Due to high absenteeism in the general Belgian workforce [55], new legislation [56] aims to facilitate a trajectory to job re-entry, and the support of the worker in RTW by multiple actors, such as the OP, is more emphasized. Preferably, the OP conducts an (informal) RTW consult before the worker resumes his/her/their job so that potential necessary work adjustments can be discussed and applied in advance. Such a ‘visit’ with the OP is a legislative right for every worker and can be requested with or without the employer’s knowledge. Formal RTW examinations are conducted after a four-week absence (certified leave) or longer. Additionally with the new legislation a re-integration trajectory can be requested by multiple parties (employee, employer, physician). Depending on Belgium’s specific regulations within labor law and health (insurance) policy and the client’s duration of work absence, OPs thus also collaborate with advising physicians of the health insurance funds for RTW trajectories. These advising physicians (AP) work as medical advisors for health insurance funds to, among others, attribute work incapacity benefits when a worker is absent for longer than one month while the employer pays the first month of disability.

### Study Design

This study is part of a larger project ‘Return to work of transgender and gender diverse people’, and was co-created with the TGD (healthcare) community to explore RTW among TGD people following steps in gender transition, identify allies, prevention measures, health promotion, and work accommodations to support the well-being and (occupational) health of TGD people. The first author adhered to the research policy of the ‘European Professional Association for Transgender Health’ (EPATH) [57] and recommendations from key figures in TGD (health) research [58, 59].

To capture the complexity of the social world [60] of TGD people, a convergent mixed method design was used with an emancipatory stance favoring underrepresented groups [61]. We collected two different data types (Quantitative and Qualitative) in parallel with each other in an interactive approach [62–64]. Quantitative data were collected through a survey and qualitative data within an ‘informed grounded theory’ approach [65] by way of semi-structured interviews. The initial quantitative data findings were used as input for collecting qualitative data in a cyclical manner. Triangulation of the data collection, and the data analysis was performed to increase the credibility of the findings. Following Creswell [62, 66], we decided not to compare the quantitative data with the qualitative, instead to convey through narrative how the results agree (converge) or provide more depth. We adhered to the ‘Strengthening the Reporting of Observational studies in Epidemiology’ (STROBE) statement [67] for quantitative findings and the ‘COnsolidated criteria for REporting Qualitative research’ (COREQ)[68] guidelines for qualitative findings while also taking the American Psychology Association’s ‘Mixed Methods Article Reporting Standards’ (MMARS) [69] into account.

### Sampling

Recruitment was based on multiple mixed methods sampling [70] from October 2020 to July 2021. Survey respondents were reached through social media, our research website (www.rtwoftransgenderpersons.com), flyer distribution at different expertise centers, waiting rooms of GAC-related services of the Ghent University Hospital, or through the help of the ‘Lesbian, gay, transgender, (gender)queer or questioning, intersex, asexual’ (LGBTQIA +) community and other networks. For the qualitative part, a purposive maximum variation (heterogeneity) sampling [71] followed by snowballing technique [72] was used. Eligibility for both the survey and the interviews entailed identifying as a TGD person who had started their social transition at work with or without GAMI in the last five years. Therefore the minimum age was restricted to twenty-three years, which allowed for the possibility of having 5 years of work experience. Participants could choose to voluntarily take part in either the survey or the interviews, or both, and receive a gift card.

### Quantitative Part: The Survey

#### Measures and Data Collection

A cross-sectional, self-administered anonymous bilingual (Dutch, French) online questionnaire was used to assess socio-demographics, work characteristics, steps in gender transition such as coming out and social transition and/or GAC (See Table 1), work absence measured in sick days and RTW, social support, turnover for reasons related to being a TGD worker, health literacy and perceived health. The survey was a combination of validated questionnaires and expert-based questions (Online Resource 2). Questioning gender identity, life situation, and GAC was based on the last national survey (2017) exploring the life of TGD people in Belgium [2] and the European Trans Health Survey (2017) [73]. The survey parts about RTW, transition at work and turnover were developed by the research team. Data were collected using Research Electronic Data Capture (REDCap), a secure, web-based software platform [74]. The questionnaire was pilot tested by five members of the TGD community, and feedback was applied before official launch.

**Table 1:** Surveyed steps in social and/or medical transition

Coming out and started social transition	adopting social roles (different from those assigned sex at birth) by changing names, pronouns, clothing, etc. regardless from medical steps
Counseling	Care and support offered by mental health professionals regarding gender exploration, stigma and discrimination, transition (e.g. social, hormonal treatment, peri-operative period)
Hormonal treatment	Hormone blockers, testosterone, estrogen
Gender-affirming surgery	Top surgery (augmentation, mastectomy), hysterectomy, ovariectomy, metoidioplasty, phalloplasty, orchiectomy, vaginoplasty, voice surgery, feminizing facial surgery, laryngeal shave surgery and other gender-affirming/related surgery

Based on J.Motmans et al. [2], Smiley et al. [73]

#### Socio-Demographic Variables, Gender Identity, and Work Characteristics

Age was questioned by birth year, education was based on International Standard Classification of Education (ISCED 2011) [75] and aggregated to levels ‘low’, ‘middle’ and ‘high’ based on Eurostat [76]. Gender identity was based on self-identification (see Online Resource 2), after which respondents could also indicate in which smaller category (transgender men, transgender women, gender diverse people consisting of genderfluid/non-binary/genderqueer/polygender people) they were to be included for data analysis purposes. Work characteristics were questioned, such as employment type, status, corporate seniority, occupational sector (which was recoded into primary to quaternary), working in a profession involving social contact, having a hierarchical position, corporate size, nature of job tasks, turnover intentions (function, employer, sector, status) in the last five years related to being a TGD person, and job satisfaction which was based on the ‘European Quality of Life Survey’ [77].

#### Work Absences and Return to Work Following Transition-Related GAC

We explored data of work absences and RTW related to social and/or medical transition (See Table 1). Respondents could check if they started social transition and the steps of their individualized transition-related GAC in the survey (yes/no) presented according to their gender identity (see Online Resource 2). Work absence (yes/no) following coming out and a step in transition-related GAC in the last five years was defined as being a (self)-certified/legitimate medical absence of at least one working day self-reported by survey respondents. Planned absences such as holidays or (unplanned) personal days (reasons other than illness or holiday, taken at the employee’s discretion) were excluded. In addition, based on the BELSTRESS III study [78] general sick leave due to medical reasons (illness, accident, or other) for the last twelve months was inquired as the total amount of sick days. Return to work was dichotomized (yes/no) and defined as going back to work (to the same or another employer) after at least one sick day following a step in their socio-medical transition and GAC.

#### Self-Perceived Health, Health Literacy, and Support at Work

Self-perceived health was measured by a five-point Likert scale ranging from ‘very good’ to ‘very bad’. Health literacy was investigated and scored through the validated (general population) short version of the European Health literacy survey questionnaire (HLS-EU-Q6) [45, 79] incorporating aspects related to mental well-being. Respondents completed this version by checking a 4-point Likert scale. Support at work during gender transition was measured for different actors on a five-point Likert scale, which was dichotomized in ‘no-little-neutral’ support and ‘(strongly) supported’ while also providing the option ‘not applicable’ that was treated as missing data.

### Data Analysis

Using IBM SPSS Statistics for Windows, Version 28, descriptive analysis was performed on the survey results. Primary outcomes and independent variables were stratified according to gender identity. Non-parametric tests (Mann–Whitney-U and Kruskal–Wallis test) were used to compare medians of continuous variables. Proportional differences and associations between categorical variables were explored using Fisher Exact test. Correlations were calculated using non-parametric Kendall-Tau-b. Cross-tabulation comparisons were done between the total sample and the RTW outcome sample. Results were interpreted as significant when *p* value < 0.05.

### Qualitative Part: In Depth-Interviews

#### Interview Guide and Data Collection

Semi-structured in-depth interviews were used to explore experiences of social transition and especially on the combination of transition-related gender-affirming medical care and (return to) work.

The semi-structured interview guide consisted of open-ended questions (Online Resource 2), developed by the research team, which were categorized in topics such as gender identification, job profile, work environment and their company’s inner workings, coming out, GAC, work absences and RTW. Attention was also given to knowledge and perception of occupational health and insurance services; role of OP and AP. Additional questions were added to the guide with a focus based on preliminary results of both survey and interviews. Due to the COVID pandemic, interviews were conducted online using video conferencing software (Zoom, MS Team) by first author (JVdC) after following a training in gender-diverse care provided by the ‘World Professional Association for Transgender Health’ (WPATH) and gender-inclusive language provided by local partners of the TGD community. The audio track of the recorded sessions was extracted and used for transcription purposes. Video material provided depth by adding emotions such as facial expressions, gestures to the transcriptions. During interviews notes were taken and verbal and non-verbal probing techniques were used to elaborate on important points made by the participants.

### Data Analysis

The audio files of the online meetings were transcribed verbatim, and transcripts were analyzed based on grounded theory using qualitative data analysis software NVivo (Version 1.4–1.6, released 2021). Initial coding was based on in vivo coding and process coding [80] through line-by-line coding to be true to vocabulary used within the TGD community. Second cycle coding consisted of focused coding [81]. The initial and second coding process was repeated by two others (DVdV, LB) on six transcriptions to ensure intercoder reliability. Through cumulative coding methods, such as pattern coding, the analytic work was synthesized [80], which was followed by freestyle memo writing [81]. Through theoretical coding (third cycle) [80] and diagramming [81] of facilitators and barriers of RTW, advanced memos were written down, and themes were constructed within the RTW process. Data saturation was reached when additional interviews generated no original views or RTW experiences and when no higher categories could be extracted from the data. Validity was based on triangulation and a member check.

### Ethics

This mixed-methods study was approved by the Ethical Committee of Ghent University (EC/2019/0863). Informed consent which included data collection, analysis and publication was given separately for the survey and/or interview. Data were saved on a Ghent University project share drive adhering to General Data Protection Regulations.

### Results

#### Quantitative Results

The questionnaire yielded 128 respondents. Of these, 125 participants answered their gender identity (GI) in detail and how to be categorized for data analysis purposes as either transgender men (TM), transgender women (TW) or gender diverse (GD) people. One hundred and nine reported on their transition-related steps in the last 5 years, 61 gave details on their RTW following steps in gender transition and this subsample had similar characteristics as the total sample. Table 2 displays socio-demographic and work characteristics of our survey respondents and, Table 3 shows gender-affirming care for the three gender identity groups along with RTW characteristics and health literacy (HL).

**Table 2:** Survey socio-demographics and work-related characteristics for total sample (n = 128) and stratified by gender identity (n = 125)

	Total sample (n = 128)		Transgender men (n = 42)		Transgender women (n = 51)		Gender diverse people (n = 32)		p
	n	%	n	%	n	%	n	%	
Age (n = 128)									< 0.01*
Median (IQR)	40 (21)		31.5 (17)*		44 (15)*		35.50 (23)		
Min–max	23–62		23–61		23–62		24–58		
Living situation (n = 94)									0.33
Living with family, friends	15	16.0	6	40.0	5	33.3	4	26.7	
Living together with partner	38	40.4	11	28.9	15	39.5	12	31.6	
Single	37	39.4	18	48.6	15	40.5	4	10.8	
Single parent	4	4.3	1	25.0	2	50.0	1	25.0	
Education^**$**^ (n = 124)									0.50
Low level	2	1.6	0	0.0	2	4.2	0	0.0	
Medium level	50	40.3	16	38.1	22	45.8	12	37.5	
High level	72	58.1	26	61.9	24	50.0	20	62.5	
Employment type (n = 123)									0.23
Full-time employment	75	61.0	28	66.7	28	58.3	17	54.8	
Part-time employment	30	24.4	11	26.2	9	18.8	10	32.3	
Other (retired/trainee/ unemployed/medical leave)	18	14.6	3	7.1	11	22.9	4	12.9	
Employment status (n = 122)									0.22
Laborer	23	18.9	6	14.3	11	23.4	5	16.1	
Employee	86	70.5	34	81.0	29	61.7	22	71.0	
Self-employed	13	10.6	0	0.0	5	10.6	1	3.2	
Interim/temporary worker/student/trainee	7	5.7	2	4.8	2	4.3	3	9.7	
Private or public sector (n = 109)									0.15
Private sector worker	78	71.6	30	75.0	32	80.0	16	59.3	
Public sector worker	31	28.4	10	25.0	8	20.0	11	40.7	
Corporate seniority (n = 121)									0.34
< 1 year	21	17.4	10	23.8	4	8.7	7	22.6	
≤ 5 years	47	38.8	16	38.1	19	41.3	11	35.5	
> 5 years	53	43.8	16	38.1	23	50.0	13	41.9	
Social contact profession (n = 120)									0.70
No	57	47.5	18	42.9	23	51.1	16	51.6	
Yes	63	52.5	24	57.1	22	48.9	15	48.4	
Hierarchical position (n = 122)									0.33
No supervising or management position	91	74.6	30	71.4	39	83.0	20	64.5	
Senior management/Senior executive	4	3.3	2	4.8	2	4.3	0	0.0	
Middle management	15	12.3	5	11.9	4	8.5	6	19.4	
Operational management or team leader (supervisor)	12	9.8	5	11.9	2	4.3	5	16.1	
Company personnel (n = 118)									0.39
≤ 50	35	29.7	15	36.6	12	27.3	8	25.8	
51—100	19	16.1	7	17.1	4	9.1	8	25.8	
101—250	10	8.5	4	9.8	3	6.8	2	6.5	
> 250	54	45.8	15	36.6	25	56.8	13	41.9	
Job tasks (n = 116)									0.11
Mainly manual tasks	10	7.8	4	9.8	3	6.8	3	10.0	
More manual tasks than intellectual tasks	15	11.7	10	24.4	2	4.5	3	10.0	
As many manual and intellectual tasks	34	26.6	9	22.0	18	40.9	7	23.3	
Mainly intellectual tasks	57	44.5	18	43.9	21	47.7	17	56.7	
Job satisfaction (n = 94)									
Median (IQR)	8 (3)		7.50* (3)		8.0* (4)		7.0 (2)		< 0.05*
Turnover related to transgender identity in last 5 years (n = 97)									0.78
No	76	78.4	27	35.5	31	40.8	18	(23.7)	
Yes	21	21.6	9	42.9	7	33.3	5	(23.8)	

Medians stratified for gender identity were compared with independent samples median test: *significantly different; Fisher exact test for variables stratified by gender identity; % column percentages; $: Low level education = primary education; Medium level = (junior) high school, 1–2 years of specialization, higher education without graduating; High level = higher education with a degree, postgraduate education

**Table 3:** Transgender health care, return to work and health literacy for total sample (*n* = 128) and stratified by gender identity (n = 125)

	Total sample (n = 128)			Trans men (n = 42)		Trans women (n = 51)		Gender diverse people (n = 32)		p-value
	n	%		n	%	n	%	n	%	
Coming out and started social transition (*n* = 109)										< 0.01*
No	15	13.8		4	9.8	2	4.9	9	33.3	
Yes	94	86.2		37	90.2	39	95.1	18	66.7	
Sick leave after coming out and starting social transition (*n* = 88)										0.14
No	75	85.2		27	79.4	31	83.8	17	100	
Yes	13	14.8		7	20.6	6	16.2	0	0	
Chosen a step in GAC (*n* = 109)										0.02*
No	7	6.4		1	2.4	1	2.4	5	18.5	
Yes	102	93.6		40	97.6	40	97.6	22	81.5	
Counts of chosen steps in GAC (*n* = 109)										< 0.01*
1 step	10	7.8		4	9.5	1	2.0	5	15.6	
2 steps	13	10.2		7	16.7	0	0.0	6	18.8	
3 steps	26	20.3		11	26.2	11	21.6	4	12.5	
≥ 4 steps	53	41.5		18	42.9	28	54.8	7	21.9	
General sick leave after steps in GAC (*n* = 94)										0.19
No	32	34.0		11	28.9	11	29.7	10	52.6	
Yes	62	66.0		27	71.1	26	70.3	9	47.4	
Sick days for all GAC (*n* = 94)										
Median (IQR)	38.0 (39.0)			32.2 (39.0)		60.0 (70.1)		24.7 (14.3)		0.21
RTW (same or other employer) (*n* = 61)										0.36
No	6	9.8		1	3.8	4	15.4	1	11.1	
Yes	55	90.2		25	96.2	22	84.6	8	88.9	
Consultation advising physician (*n* = 97)										
None	79	84.0		32	88.9	28	80.0	19	82.6	0.82
1	10	10.6		3	8.3	4	11.4	3	13.0	
≥ 2	5	5.3		1	2.8	3	8.6	1	4.3	
Consultation occupational physician (*n* = 97)										
None	64	66.0		26	72.2	22	57.9	16	69.6	0.73
1	21	21.6		6	16.7	10	26.3	5	21.7	
≥ 2	12	12.4		4	11.1	6	15.8	2	8.7	
Advice from occupational physician (*n* = 33)										0.41
No help	24	72.7		8	80.0	10	62.5	6	85.7	
Advice or work adjustments	9	27.3		2	20.0	6	37.5	1	14.3	
Health perception (*n* = 95)										0.04*
Very good health	14	14.7		2	5.6	10	26.3	2	9.5	
Good health	49	51.6		22	61.1	18	47.4	9	42.9	
Mediocre health	24	25.3		7	19.4	7	18.4	10	47.6	
Bad health	7	7.4		4	11.1	3	7.9	0	0.0	
Health literacy level (*n* = 78)										0.73
(Likely) inadequate	8	10.3		2	6.7	3	10.0	3	16.7	
(Likely) problematic	27	34.6		11	36.7	9	30.0	7	38.9	
(Likely) sufficient	43	55.1		17	56.7	18	60.0	8	44.4	
Health literacy score (*n* = 78)										
Median (IQR)	3.0 (1.0)			3.0 (0.88)		3.25 (1.21)		2.67 (0.88)		0.21

%: column percentages; p: *p* value; *significant; social transition: coming out (name, pronouns) and adopting gender roles different from those assigned at birth regardless of medical interventions;

*GAC* gender-affirming care that is transition-related comprised of counseling, hormonal therapy, gender-affirming surgery; *GD people* gender diverse people; *RTW* return to work following GAC; *IQR* interquartile range; proportions were compared by Fisher Exact test; medians compared by way of Kruskal–Wallis test

#### Transition (-Related) Gender-Affirming Care

From the total sample, most respondents had started their social transition (86.2%), but GD people disclosed their gender identity significantly less. The majority (93.6%) undertook a step within transition-related GAC in the last 5 years (see Table 3) but this was less often for GD people (taking 0–2 steps) while 55% of TW reported ≥ four steps. Most participants (72.6%) also considered their transition as still an ongoing process. Nearly all respondents (96.2%, *n* = 104) indicated the use of gender-affirming hormonal therapy (96.2%, *n* = 104), while top surgery (feminization, *n* = 22 and masculinization, *n* = 14) and genital GAS (vaginoplasty, *n* = 15; orchiectomy *n* = 10; hysterectomy *n* = 10; *n* = 8, phalloplasty) were the most reported surgical procedures. Top surgery was the most frequent (45.3%) surgical step of transgender men in our total sample (*n* = 128) while breast augmentation as well as vaginoplasty were equally (29.4%) reported by transgender women. Only a handful of GD individuals reported undergoing GAS (mostly top surgery, *n* = 5) whereas hormonal therapy (*n* = 16) was a more common shared medical decision (50%).

#### Work Absence and Return to Work After Transition-Related Gender-Affirming Care

General sick leave due to medical reasons (illness, accident, or other) in the past twelve months (2020–2021) was on average two weeks (median 14.0 days, IQR 40.0) in our survey sample. Most respondents from our subsample on steps in gender transition (*n* = 109, see Table 2), indicated being on sick leave following their coming out (during social transition) and after GAS. After one or combined steps in transition-related gender-affirming interventions, 66% (*n* = 62) of participants in general (*n* = 94), reported having been absent from work, with a total median of 38 days (39 IQR) of medical leave. The total median work absence (sick days) for GAC was the highest for TW, followed by TM and the lowest for GD people but these differences were not significant (*p* 0.214) (see Table 3). Visits with the AP of their health insurance fund, in the context of absenteeism during gender transition, were only reported by 18% of participants. After their coming out and steps in GAC, *the majority (90.2%) returned to work* at the same workplace (72.1%) or resumed working at another employer (18.0%). Stratification by gender identity was not significant (*p* = 0.361) but higher non-RTW was seen in TW.

#### Health Perceptions, Health Literacy and Support

Sixty-six percent perceived their health to be good to very good, but GD people had a significantly (*p* = 0.04) more mediocre perception of their health. The average HL score was low and only 55.1% of participants displayed a sufficient HL level (see additional Fig. 1 in ‘Online Resource 3’). No significant differences were, however, observed for gender identity (see Table 3) for HL but GD people scored the worst. Despite this, nearly 90% TGD individuals received some form of support (such as practical, emotional, moral, financial, or administrative) during their transition while at work (See ‘Online Resource 3'). Although there were no significant proportional differences (*p* = 0.09), TM and TW reported receiving equal levels of support, whereas GD individuals, in general, reported receiving less support. Additional information in ‘Online Resource 3’ indicates that certain individuals or services were, however, not accessible in several companies. Respondents received the most support from their coworkers (*n* = 64; 71.9%), supervisor (*n* = 60; 69.0%), and work confidant (*n* = 41; 66.1%), with no significant differences based on gender identity. However, less than half of TGD individuals felt supported by the human resources department. Interestingly, TW were significantly (*p* = 0.045) more likely to receive support from the human resources department than TM and GD individuals. One-third of the participants reported receiving support from their occupational physician, this was particularly evident in the case of TW (*p* = 0.006). Furthermore, a mere 22% had a single consultation with their company OP during their sociomedical transition regarding a possible OHS directed RTW process or general health surveillance. During this visit, 72.3% of respondents did not receive any helpful advice or work adjustments. However, for those who did receive advice or accommodations, examples included graded return to work, reduced working hours, alternative work tasks, or infrastructural accommodations like a separate changing room. Some respondents even requested medical discharge.

### Qualitative Data

The diverse range of participants’ background characteristics are summarized in Table 4. All participants shared their personal gender identification realization and journey, their experience of coming out and transitioning at work, their gender transition-related absence period and RTW. During the analysis of their input (views and experiences), four distinct themes emerged regarding the support and prevention strategies for TGD individuals in the workplace: (1) the need for and access to information; (2) the need of having educated allies; (3) the influence of their occupational position; (4) navigating the balance between work, life and GAC.

**Table 4:** Characteristics of interview participants (*n* = 20)

Pseudonym	Duration (min)	Self-perceived gender identity	Age Group (years)	Education	Employment type	Corporate seniority
Angela	248	Trans feminine non-binary person	40–49	High level	Self-employed as 2nd activity^a^	2
Arthur	85	(Trans) boy	20–29	High level	Employee^£^	2
Barbie	143	Trans woman	50–59	High level	Government employee^a^	27
Berg	145	Non-binary person	30–39	High level	Employee^a^	5
Elle	125	(Trans) woman	30–39	High level	Employee	16
Els	145	Non-binary person	40–49	High level	Government employee	5
Fleur	143	(Trans) woman	30–39	High level	Government employee^S^	11
Lily	115	Trans woman	50–59	High level	Employee	35
Luna	72	(Trans) woman	30–39	High level	Employee	4
Megan	104	(Trans) woman	40–49	Middle level	Laborer	16
Merel	160	Bigender, non-binary person	30–39	Middle level	Employee	1.5
Mikal	63	(Trans) woman	40–49	Middle level	Laborer	15
Miro	113	Trans masculine non-binary person	40–49	High level	Employee	21
Nathan	101	Trans masculine non-binary person	20–29	Middle level	Employee^£,S^	1
Pierre	112	(Trans) man	30–39	High level	Employee^£^	5
Ranma	96	(Trans) woman	40–49	High level	Laborer	15
Saar	156	(Trans) woman	50–59	Middle level	Self-employed^S,M^	15
Taylor	116	(Trans) woman	30–39	High level	Employee^S,M^	3
Victor	91	(Trans) man	20–29	High level	Employee^S^	< 1
Will	135	(Trans) man	20–29	High level	Employee	< 1
Total (mean)	2472 (123)					

^a^Became recently unemployed during their GI transition and receive benefits, ^£^part-timers, ^S^supervising function, ^M^management position

### Theme 1: The Need for Information and Navigating the Work-Related Health Systems

After analyzing the interviews, it was clear that all participants believed having information about work absences and returning to work was important. This includes knowing how to navigate through different occupational health and social insurance systems, especially as a vulnerable group with known barriers to care.

### Subtheme 1.1: Central Point of Contact

Participants needed guidance on GAC-related occupational (health) implications, medical leave regulations and RTW and felt the lack of it, more than for other health-related absences (non-GAC), from health professionals as well as from company stakeholders. A wish for a central point of contact at work was mentioned, who was to be equipped with certain knowledge ranging from information about gender, transition at work, work absences and -resumption dependent on participant's company structure and size or knew the right referral*.*

“Because what I missed was actually one central person, I could turn to… So, you don't have to go ‘shopping’ and then have to repeat the whole explanation.” (Miro)

“Really, for trans people, it’s the way they will be received by their coworkers that makes going back to work difficult and having someone who is in charge of these questions (on gender identity and transition) in a structured way...that would be beneficial, also for coworkers” (Arthur)

The affiliation of this central point of contact with the LGBTQIA + community or experience with the subject and the interviewee’s perception of the organization's trans friendliness could either help or hinder their willingness to ask questions and seek help. According to participants, access to that central point should be transparent by way of advertising in public rooms at work (e.g., cafeteria, hallways) with name, location, and pictures (occasionally also with pronouns).

### Subtheme 1.2: Occupational Health Literacy

Participants demonstrated a strong willingness and ability to search for information about transition-related GAC, comprehend it, and put it into practice. However, it should be noted that their ‘health literacy’ was limited to that specific domain and did not extend to the aspects of GAC that pertain to their work. They were often not aware of their professional risks, their company’s structure, and affiliations with an occupational health service (OHS), labor regulations and trade union delegations. They rarely knew of occupational health and safety management or preventive measures for well-being at work. The role of OHS was misunderstood and the preventive medical examinations by an occupational physician (OP) were skeptically evaluated and labeled as superfluous. Participants with a good awareness of their job demands, occupational risks and with regular contacts with OHS were regarded as “occupational health literate” by first author and interviewer (JVdC). They told a more comprehensive narrative on the OHS’ role and possible support. However, although these participants were mainly in a higher occupational position at work (seniority, hierarchy level) and portrayed themselves as more empowered or worked closely with HR, specific knowledge about the RTW process, all actors it entailed and procedures benefitting the workers were still (partially) lacking, apart from two individuals.

**“**We have our own internal service for protection at work, but you rarely hear about or see anything from them. The only thing those people do is occasionally visit some buildings to see if there is no asbestos or something else…. I am convinced occupational physicians can be useful for work accommodations, but at our workplace, I think these doctors have no affinity with transgender people.” (Els)

### Subtheme 1.3: Social Insurance Literacy: ‘They Always Assume you Know it All’

The analysis showed participants experienced difficulties having to fill in ‘elusive’ forms and submit documents such as a medical certificate to the health insurance fund while already having notified their employer. Frustrations arose while already having enough things on their mind, as not having this knowledge led to financial distress such as late reimbursements of their (sickness) benefits. Most did not take initiative upfront to search for information about social health security or consult websites of health insurance funds, showing a lack of ‘social insurance literacy’. Participants felt they should be better informed by health professionals and advising physicians concerning insurance and benefits. In addition, participants believed the employer bore responsibility to inform about such procedures to understand work absence regulations and submit the correct paperwork. Interviewees emphasized such knowledge should not be taken for granted by employers, human resources, and health professionals.

“What really upset me, I was put onto sickness benefits for my “Facial Feminization Surgery”, as I understand you then receive your wage from a health insurance fund. But honestly, I had no prior knowledge of that. And suddenly my wage was not deposited… I was fined too; they didn't pay the full amount because they were notified too late. We should know about that, but nobody cautioned me about it. And I had clearly communicated to HR that I was going on sick leave but they didn’t explain anything. They always assume you know it all.“ (Fleur)

### Theme 2: The Need for Transgender Allies as Mediators

In addition to HR managers, supervisors, and colleagues, OHS advisors were perceived as potential allies for TGD individuals. They could act as mediators during the transition process at work, provide information, and play a specific role in the return to work process, such as facilitating a graded work resumption plan.

### Subtheme 2.1: The Occupational Physician as the Mediator

The analysis showed a distinction between participants who knew of the occupational physician's existence and those who did not. Participants who did not know the OP had never gone to a consultation even when their company was affiliated with an OHS or had only seen the OP in a periodic capacity and considered this to be the main purpose. Those who knew the OP had different expectations towards the role of this health professional based on their previous experiences. After informing participants (by JVdC), who were unaware of the role of the OP or were mistaken with that of the advising or another physician, some believed these health professionals to be a great potential ally and declared they would have used their service. They were shocked over the lack of communication from their company about possible support from OHS and OP.

“Just knowing there is an occupational physician would have done a lot for me. <<laughs>>...And what I have always missed is uhm...Indeed being able to visit someone who can help me and also uhm I couldn't indicate what I needed. That was very difficult for me… I mean a doctor from the hospital isn't going to tell you all what you can do and what you can't do at work and stuff, and for me that was something very difficult to indicate to my supervisor and coworkers." (Victor)

Participants felt the aspect of professional secrecy was an important part of the trust they put in the OP. However, TGD people expect OPs to be well informed about GAC and for some it became clear their OP was sometimes not sufficiently prepared for TGD healthcare or aftercare. Participants felt biomedical aspects, in general, were predominant during OP visits. They downgraded some of the technical examinations according to their job risks. Having knowledgeable OP about transgender health aspects, on the other hand, proved to be very validating for TGD people. Interviewees emphasized however that OPs can only be useful being aware of the company's policies, by visiting the workplace for a tailored approach to suggest adequate work adjustments, to normalize GAC and TGD people’s needs and always act respectfully. Furthermore, when the company’s hierarchy was seen treating OHS advice in a step-motherly way, this downgraded the possible impact of the OP’s support.

### Subtheme 2.2: The Occupational Health Service as a Support System

Participants considered the occupational health service (OHS) as an added value to transitioning at work. Although some were skeptical about the impact on the management level, most saw several ways OHS could support TGD workers if they were up to date on GAC and aware of its significance for a diverse and inclusive work climate.

As to communication about OHS, there were mixed experiences ranging from being notified through work to never even hearing about it. Some stated they would have made different decisions during transition at work had they been aware of OHS. The OHS was thus perceived as a potential ally in being a central point of information helping with disclosure at work, providing psychosocial and practical support and educating companies in gender diversity and transition at work.

“I think communication about OHS could help for different things…, if I have a bad day at work, they will very quickly attribute it to my transition...not cool...and what if you really have a problem concerning your transition at work, to whom should you go for help and support?” (Victor)

In addition, interviewees stressed the significance of having a multidisciplinary approach when it comes to the OHS in addressing the unique needs of TGD individuals, as well as the specific requirements of the company and industry and the collaboration with GAC professionals. Furthermore, the interviewees highlighted the crucial role of transparent and easily accessible communication from the company regarding OHS.

**“**I found it very strange that the gender team could not actually refer me to a person at work who could have helped me! So, I would say more bridges can be built between healthcare services.” (Nathan)

### Subtheme 2.3: Graded RTW and RTW Plan

One of our earliest interviewees, a GD person, mentioned the principle of graded RTW and the need to discuss the RTW process well beforehand with ample room for individualism and expressing needs. Therefore, graded RTW was further explored during the following interviews. It was acknowledged that these needs are not always easy to voice, and attention should be given to thoroughly inquire about them.

“I know nów if I ever have another surgery... I should mention my limits in advance… There is a system of progressive resumption of work... I would have preferred this... Because you can't really estimate the impact... In retrospect, you think it *seems so obvious,* but I didn't even think of asking about it, and nobody at work even thought about suggesting it.” (Miro)

Although participants felt a stepwise and preparatory RTW plan was a helpful approach, it was also pointed out, by a GD individual, as doing things half-heartedly. In addition, individuals in a hierarchical position who had the ability to determine their own schedule for taking medical leave and returning to work stated that a return to work plan was not necessary.

### Theme 3: The Position in the Work Environment

Participants, at one point or another, had strong convictions about their occupational position and how this influenced their perception of feedback and support they would receive on their coming out, their decision to transition at the same workplace, and their ability to take a stand in their transition plan. This occupational position varied between having a certain seniority, their ranking and status within the hierarchy, and the upmake of their contract type.

### Subtheme 3.1: Seniority

Interviewees were convinced high seniority guaranteed a better foundation, a better social relationship with colleagues, and mutual respect which they relied on for gender affirmation and support. Seniority played a role in the choice of transitioning at the same workplace. Apart from financial reasons, the build-up of seniority could also be the reason for delaying disclosure. TGD people imagined the difficulty of revealing the ‘real me’ to colleagues who had known them as another identity for so many years and understood that, for some, turnover with disclosing at a new workplace or without could be more accessible.

“I don't think my position has determined anything, but my seniority did enormously. And also, the amount of respect you have built up towards people, and they know who you are, what you stand for, and what you can do for them.” (Elle)

Seniority was understood as a way to obtain more flexibility in company policy, negotiations of the transition plan, and being absent in light of work schedules and demands. TGD people with seniority received more respect from the hierarchy as valuable workers and experienced the necessary feeling of job security during medical leave and when returning to work. Participants recognized chances were high the situation would be less flexible if they were 'a mere worker who just had started'*,* and in that case, an existing trans-inclusive policy would be desirable alongside the help of allies, such as OHS, legal parties and union.

### Subtheme 3.2: Ranking

A higher ranking in hierarchy gave the flexibility to manage their own work schedule around GAC appointments. Depending on their ranking, they had the autonomy to participate in and make their own decisions regarding their coming out and transitioning at work but also informal arrangements in their RTW. Due to their higher ranking, there was never a genuine concern about how team members or subordinates would react. There was an implicit sense of empowerment wherein there was no need for an exemplary role of a manager to create an inclusive gender-affirming work climate.

### Theme 4: Maintaining a Good Work-Life-GAC Balance

Mainly when not being in a high-level position at work, participants found it difficult to manage their career and GAC needs. Agreements made with the employer, and their flexibility, greatly influenced the gradation of navigation within gender-affirming healthcare. Furthermore, personal coping strategies and views on work ethics, financial situation, and the ability to find work-related support (OHS, colleagues, union, etc.) largely affected work-life-GAC balance.

### Subtheme 4.1: Influence of Employer/Management

When employers tended to voice their dissatisfaction of regulated work absences, this sparked a fear in TGD employees which lead to overcompensation behavior at work and the excessive use of regular vacation days instead of medical days overtly leading to strained work-life balance. Participants sometimes managed their GAC appointments by turning their entire daily schedule upside down to work their obligated hours. All agreed that hospital-related care especially, where there is not much leeway regarding consultations, proved quite challenging in rigid work structures or in lack of good relationships with their hierarchy.

“I think that a certain flexibility in terms of medical appointments is quite important. If there are ways to arrange work schedules, that would be beneficial.” (Arthur)

Moreover, participants were always dependent on the waiting list of the GAC services which proved to be an extra burden/stress factor while trying to manage a smooth transition at work. They are also frequently facing a stigmatized healthcare climate because “as a trans person you are surrounded by caregivers who are all cisgender, when healthcare is tailored by people who are not part of the target group, then there will always be some blind spots.” (Nathan).

Generally, the management of medical appointments was also found to be more burdensome than surgery itself due to their frequency and the need for scattered and/or combination of use of sick leave and vacation days or personal days.

“As part of my transition, I have 240 appointments a year to manage alongside my full-time job, which means I often miss meetings and take a lot of leave. Basically, I believe that my work has not been significantly impacted by the operations themselves. My scattered absence due to all the appointments in between has had a greater influence on my work, more so than a block of consecutive absence from my operations.”(Fleur)

### Subtheme 4.2: Coping Strategies: Becoming the People Pleaser

Participants who tended to rely on avoidance coping, had a negative perception of company policies and who lacked initiative or held more negative worldviews, tended to experience more ‘harsh’ difficulties during their transition at work. So, while being a lifesaver, GAC also proved an additional significant stressor in their lives. One participant, a TW, even tolerated an official sanction at work for being absent for fear of losing her job and chose stability at her current employment to be financially able to afford her GAC.

“I kept on going and going, no matter how difficult, because of the financial pressure.” (Elle)

Participants also noticed an evolution in the reception of GAC at work, as transition at work progressed, the support by coworkers and the hierarchy for some TGD people diminished which resulted in their overcompensation behavior. While navigating their career and GAC, TGD people always took into consideration not to burden their coworkers to avoid frustrations and keep their 'goodwill and support’. Colleagues and management were consulted when planning GAC steps, and more invasive ones were opted for especially in periods with less work pressure for coworkers, albeit not necessarily for themselves.

“I am having a minor operation. I can take sick leave, but I have also agreed that we will do a bit of both if I can use vacation days. I suggested that, because I also understand that losing an employee can create a difficult situation for the company too. I am trying to find a middle ground somewhere.” (Ranma).

### Subtheme 4.3: Transition-Regulated Leave

The concept of regulated transition vacation days surfaced during the interviews and was seen as a ‘trial policy’ which only existed for a small part of the public sector workforce in the Northern part of Belgium. Participants viewed this as a manifest improvement in their lives, as a strong symbol of support for transgender rights and as an inclusive work environment. The concept was also compared with the regulations of medical visits during pregnancy and with childbirth leave. However, participants found there was still room for improvement in the policy, such as elaborating its use beyond outpatient care and foregoing the request of proof as the use of certificates is not applicable in all GAC (e.g., speech therapy, electrolysis).

“I think transition leave is a good idea. Because transition is still a psychological and medical process which is quite heavy. It is about putting a lot of things in place. There are a lot of types of leave that exist in the organization. For myself, I think it would be a good idea if transition leave was already an established tradition”. (Arthur).

### Integrated Results

Overall, our survey results are corroborated by our qualitative findings, which provide deeper context to possible allyship for TGD people during social and/or medical transition. After a step in their transition accompanied by work absence, 90.2% of our survey participants and 85% of the interviewees returned to work, and nearly all to the same employer*.* Few participants in both samples took a career break during their transition or left their job. For interviewees, changed interests towards job content or seeking further education, age or accompanying health issues played a role in their turnover intention. Although RTW is high following GAC, our quantitative and qualitative results indicate a high need for more practical and informational support. This could raise the feeling of empowerment in TGD workers and lower barriers to navigate occupational and social health services. In part, minding the vulnerability of stigma and psychosocial challenges which influence their quality of life, employers should be more attentive towards work-life balance of TGD personnel and recognize the significance of occupational status. An inclusive company culture could provide more fluid guidance between health-related services and the workplace. This means that OHS should work on increasing their visibility and accessibility while also promoting a current understanding of GAC and the health effects of gender minority stress.

## Discussion

This paper reports the results of the first mixed-methods study to explore RTW outcomes of TGD people by conducting a survey and exploring experiences of social and/or medical transition. Through this study we aimed to contribute to the literature and practice of supporting TGD people at work, especially regarding work absences and job resumption, of which a clear gap of knowledge [37] exists.

Key findings are that coming out and transition-related GAC can result in temporary work incapacity and while several types of work absences come into play, the majority go back to work (to the same employer). Still, occupational health care navigation, support, and managing a work-life balance were subject to low health literacy, stigma, and the high frequency of GAC visits. Our results may nonetheless indicate that their workplaces have a positive and inclusive working climate or underline the well-known Belgian tendency for low turnover [82] while the challenging socio-economic circumstances faced by TGD people in light of the COVID pandemic [83] should not be ignored*.*

Returning to work following steps in gender transition is, however, a complex process, affected by multiple factors that are entangled in social dynamics in line with Tjulin A. and MacEachen E. 2016 [36]. The barriers and facilitators that were identified in this study, influenced by gender minority stress relate to: individual factors (e.g. personalized GAC journeys and work absences according to gender identity, health- and social insurance literacy), the (occupational) health care system and regulations (e.g. information, policies on RTW, the OP as a TGD ally, being a health literate organization) as well as work-related factors (e.g. adjustments, occupational position, social support).

Comparing work absences, the total general absenteeism of our participants is similar to that of the general Belgian population in 2020 [55] and with the sample of TGD individuals (without surgical wish) from P. Vennix [11]. However, these positive comparisons should be interpreted with caution in light of the COVID pandemic as most TGD people in our survey considered their transition as ongoing and Koehler et al. [83] reported that 50% of TGD people experienced restrictions (e.g. postponed, canceled, etcetera) to GAC during the pandemic. It is challenging to compare GAC-related work absences due to limited research [37] and differences in design and gender diversity in an earlier study [11], while our study had a more balanced sample and reported more detail of GAC. Moreover, as indicated by Oorthuys et al. [48], receiving GAC can be burdensome and can affect the work-life balance, as emphasized by our interviewees, leading to several types of work absences of which our survey only reports sick days. When comparing the latter, TM and GD individuals’ sick leave was similar as their personalized journey did not always include surgery as gender-affirming hormonal therapy was more reported by them. This aligns with previous research [2, 4, 84–86] and was also emphasized by those interviewed, who noted the significant impact it had on their lives. These results, however, underline the psychosocial challenges that contribute to work incapacities, such as stigma and discrimination during social transition which is in line with literature [87]. Additionally the higher total GAC-absence of TW could partly be attributed to more need of invasive feminizing gender-affirming surgical procedures (e.g., vaginoplasty) [1] which is in line with previous studies [4, 85] and may require a longer hospital stay [88, 89] and recovery period based on clinical practice. Subsequently, higher (not significant) non-RTW was also seen in TW, but the small sample size limits our ability to draw definitive conclusions about the impact of gender identity on work resumption. RTW experiences were regardless generally positive, but RTW proceedings were often less strict for those in ‘binary gender transition’ which participants attributed to the current social climate which is line with general work experiences reported in the last national survey [2].

Other individual/personal factors refer to a rather insufficient level of health literacy (HL) which is lower than in the general population assessment (66.6%) [44]. While in this national study [44] men were more likely to display sufficient HL, this was the case for TW in our survey. They also reported a better perception of health and more corporate seniority, known factors in HL studies [90, 91] which may partially account for these results. The worse level of HL of TM and especially GD people can be attributed to their lower socio-economic status and health which is in line with those reported in the literature on experienced microaggressions [17] and discrimination in healthcare [7, 84]. Although TGD people perceive they have knowledge and high capabilities in finding GAC-related information, they struggle with accessing and navigating the occupational health and social insurance systems to find out about sick leave regulation and RTW procedures*.* These results mirror a focus group study [92] that advocates the importance of HL within GAC and attributed low HL levels to barriers created by health professionals. This may in part explain barriers to retrieve and manage information on specific RTW issues that were encountered in our study. Moreover, the bureaucratic processes of (access to) occupational health (services), RTW and periodic visits based on occupational exposure and risks, are similar to reported difficulties in a social insurance literacy review [93]. People with good occupational health literacy who understand their job demands and risks, manage to navigate work regulations and are able to find the right person or service for information. Having these skills, TGD people are more confident and empowered to voice their views, contact OHS and OP for support during transition and to create a tailored-made RTW plan. Additionally, TGD people with insufficient social insurance literacy, who cannot comply with the administrative procedures to receive their sickness benefits in a proper way, face insecurity and loss of trust in the employer and health insurance professionals. These feelings can act as a barrier to a smooth RTW at the same workplace, as both the worker and the employer/human resources have assumptions, which is in accordance with the HL model of Soellner et al. [42] (see ‘Online Resource 1’). Each party presumes either that the knowledge is present, or the information should have been given and places the ‘responsibility’ with the other. Health professionals also tend to overestimate their client’s HL [94] and presume they are already informed about medical absence procedures by the employer or have checked it themselves. Therefore, health professionals should always assess this in unambiguous language at the end of a consultation and the employer/human resources should always check if every employee is familiar with the entire procedure. As such, ‘tailoring support works by empowering' on all levels.

Regarding factors related to healthcare services and systems, the survey and interviews conducted both indicate that TGD individuals have limited knowledge about the role and availability of OHS and OPs. This lack of understanding can negatively impact this already vulnerable group of workers, who experience additional stressors due to being a gender minority and the resulting health effects. It is unclear whether the absence of certain services at work is solely responsible for the issue at hand. Despite current Belgian legislation and practice, general problems of visibility of occupational medicine and perceptions of OHS have been highlighted in the general workforce [95–99] but this lack of visibility, however, may be more pronounced in the TGD workforce as they already experience barriers to care and problems in navigating health services [4]. Lack of communication between employees and their employers regarding these services may additionally decrease the help-seeking behavior already impacted by social pressures in the workplace. If properly equipped with knowledge on GAC, gender-inclusive language and workplace policies, the OHS and OP can be powerful allies. Workplace accommodations and tailored RTW planning by OP such as stepwise return, working flexible hours, doing less strenuous tasks etcetera) help to facilitate RTW. Not receiving practical recommendations, previous bad experiences due to a cisgender (occupational) health care setting and poor perception of the impact of OPs and OHS were expressed by the participants as hampering the RTW process. These findings support earlier studies [7, 84, 92, 100] showing the importance of knowing support inside and outside the workplace along with the usefulness of OHS during transition as well as OPs’ role in sustainable RTW. The need to raise the awareness and education of potential allies on GAC was stressed by participants, which is in line with a recent Greek qualitative study [101]. To encourage work resumption, organizations should also inform their employees on OHS-related possibilities with communication that is more attentive to TGD workers. Additionally, occupational health professionals, from their part, could benefit from education on steps in gender transition as well as psychosocial and mental health as earlier research [102, 103] has shown this to be necessary and beneficial. Thus to promote successful RTW practices of TGD people, investments should be made by the OHS along with the work environment and the health sector to raise the (occupational and gender-affirming care) health literacy of all support actors involved as advocated by earlier work [35].

Work-related factors, such as having a good occupational position, and better cooperation with management, make it more feasible to resume work through graded RTW programs and more flexible adjustments. When TGD people do not have the benefits of high rank or seniority, the aspect of job security weighs in and expectations of negative reception of disclosure and transitioning at work may discourage them from disclosing and/or choose GAC. The effect of an unsupportive employer and colleagues sometimes hastened the time to RTW or served as an immense demotivator resulting in job turnover. In addition, the influence of management as well as the personality of the TGD employee affected the work-life balance which in extension was viewed by participants as a prelude to their RTW. Positive views on RTW often, along with coworker and supervisor support, went hand in hand with less stress and more motivation to go back to work. Negative worker-employer relations and less favorable coping styles could lead to ‘being the extra-productive worker’ and, due to the repetitiveness of GAC visits and needing time off from work, using personal vacation instead of medically certified leave ending in a strained work-life balance. These findings of stressful GAC management are in line with the literature [48]. Legislative support, such as medical absences and transition-related paid absence, can therefore inherently be a positive influence on the work-life-GAC balance apart from personal views. Worries and feelings of discouragement emphasize the need for good social relations and psychosocial support from allies and third parties, which has also been suggested in other studies [2, 10, 23, 27, 28].

## Strengths and Limitations

To our knowledge, this is the first mixed-methods study on facilitators and barriers to RTW of TGD people following gender identity disclosure and/or transition-related GAC. With this first exploratory study, which was co-designed with and pilot tested by the TGD (healthcare) community, we have shown high RTW in a vulnerable population that seems to be less known to occupational physicians. We also highlighted the complexity of job re-entry by the unique stressor of gender minority stress, which affects (occupational) health literacy that was stressed by low measured health literacy levels. Furthermore, we revealed the added burden of managing a work-life balance while seeking GAC and adopting overcompensating coping behavior for which additional support through legislation is left wanting.

However, this study which was co-designed by the TGD (healthcare) community, may be limited by the focus of our study aims and be seen as ‘othering or medicalization’. This is certainly not the intent of the authors as they were informed by lack of data on work absences and RTW following transition-related GAC. Furthermore, although based on earlier European and Belgian studies and the possibility of free text, not all transition-related GAC was accounted for, such as physiotherapy, while counseling (e.g. gender exploring, transition-related) was presented as a general question not accounting for the number of sessions nor for the relatedness with the transition (e.g. other mental health aspects). The attribution of work absence (sick days) to GAC was based on self-report and this did not account for short-term work absences (hourly, holiday, unpaid days) often used for ambulant care, the latter which as reported in theme four seemed to affect the quality of life of interviewees. Additionally, while survey parts about GAC were based on previous research in TGD populations in Belgium and Europe, the health literacy questionnaire was only validated in European general population studies, however it remains a good concise instrument to use in research that has another main focus or objective such as our current study. Furthermore, although based on previous Belgian studies, the general absenteeism exploration, might have been interpreted slightly ambiguously by participants due to part of the wording used (e.g. ‘other medical reason’).

Although the survey's small sample size limits our study as well, we added value by conducting interviews until data saturation. As such, we analyzed in both parts rich data of a heterogeneous group of TGD people with a substantial number of GD participants who had also undertaken steps in GAC. The latter underlines the importance of support and further tailored guidance by health professionals of this group. Additionally, it is not uncommon to have small sample sizes in TGD research, as participants are often recruited through clinics, online, or at conferences. However, our data collection took place during the COVID pandemic. Our RTW subsample was also limited as not all respondents revealed their gender-affirming interventions and RTW status. This is understandable as a transition process is a deeply personal and private journey, despite the survey being anonymous. Interview participants were, however, much more forward about their experiences which can be attributed to the more comfortable format. The use of online interviews also facilitated the expression of participant’s opinions, which enabled us to better understand the survey findings.

In terms of qualitative data analysis, which was based on informed grounded theory, coding reliability was ensured by double coding and deliberation. Although grounded theory is based on data buildup, pre-existing knowledge cannot be erased as the main author conducted a prior systematic review [37] on the matter. The integration of results was therefore supported by continuous discussion between the researchers and adjustments when deemed necessary, to improve the analysis's depth and quality. A limitation of the qualitative part of our study is that an attempted member check resulted in meager direct feedback which increases bias, however, in an informal capacity our study results with different participants and in the general TGD community were well received. Another limitation is that our interview respondents were predominantly white, which creates bias on the level of intersectionality. Regarding the aspect of insider/outsider research, the authors are professionals with expertise in occupational health and GAC and are cisgender people, apart from two LGBTQIA + authors. Throughout the process of data collection and analysis, we have been mindful of our perspectives and worked diligently to accurately convey the participants' views.

## Implications for Practice and Future Research

As the TGD community continues to grow, the Standards of Care [1] still lack information on work resumption in their aftercare section. Follow-up of TGD people needs interdisciplinary communication, without disregard of the occupational physician, and should be focused on client empowerment, by raising their awareness and knowledge in the knowhow of our health system including the RTW process.

Apart from raising awareness and educating future health professionals in general, the current generation of OPs should expand their knowledge on GAC, improve their communication and appraisal skills to lower barriers to care and as such make part of a health literate structure and be more mindful during the periodic health surveillance of workers about the health effects of gender minority stress. As OPs are also getting more involved in psychosocial issues and the mental health of the workforce, future research should aim to explore further how those aspects are being addressed in a more vulnerable population subjected to stigma and discrimination, and how this can be improved. The aspect of becoming an inclusive and health literate work environment, managing the work-life balance of TGD workers undertaking steps in gender transition should be explored further by OHS in a participatory (interventions) studies with all stakeholders such as the TGD community and GAC professionals, employers and policy-makers to substantiate supportive policy changes such as transition-related leave. As rehabilitation legislation is very nation-specific, these studies should be conducted on a regional level.

## Conclusions

This is the first study to investigate work absences and -resumption of transgender and gender diverse people following transition-related gender-affirming care using a mixed-methods approach. Additionally, their level of health literacy was measured for the first time as recorded in research to our knowledge.

The RTW rate in our Belgian TGD population was high and, although it stems from a small sample, it is a motivating message towards corporate actors to invest in a TGD friendly inclusive work environment. Nevertheless, a need for better information and access to health care and insurance systems is illustrated, along with a strained work-life balance subject to socially induced maladaptive coping and gender minority stress, and thus an individualized RTW plan that addresses potential barriers for re-employment would be beneficial. Most of these factors are similar to RTW research of cisgender people and should relate the overall message to not wrongfully label TGD people and to consider gender-affirming care as standard within the (occupational) health care.

## Supplementary Information

Below is the link to the electronic supplementary material.

Supplementary file1 (PDF 417 KB)—Supplementary information 1 (‘Online Resource 1’) is a term of reference and overview of two health literacy models (Figures 1-3)

Supplementary file2 (PDF 216 KB)—Supplementary information 2 (‘Online Resource 2’) contains examples from the questionnaire and interview guide.

Supplementary file3 (PDF 304 KB)—Supplementary Information 2 (‘Online Resource 3) contains detailed health literacy and support at work results (Figure 4-5, Table 5).

## Data Availability

The data generated and analyzed during the current study are governed by the legal provisions of Ghent University, Belgium. They are not publicly available owing to the sensitive and personal nature of the data, moreover the datasets constitute an excerpt of research in progress.

## References

[1] Coleman E, Radix AE, Bouman WP, Brown GR, de Vries ALC, Deutsch MB, et al. Standards of care for the health of transgender and gender diverse people, version 8. Int J Transgender Health. 2022. 10.1080/26895269.2022.2100644. 10.1080/26895269.2022.2100644 PMC9553112 36238954

[2] Motmans Joz, Wyverkens E, Defreyne J. Being transgender in Belgium: 10 years later. Brussels: Institute for the Equality of Women and Men; 2017. p. 22–64.

[3] Hembree WC, Cohen-Kettenis PT, Gooren L, Hannema SE, Meyer WJ, Hassan Murad M, et al. Endocrine treatment of gender-dysphoric/gender-incongruent persons: an Endocrine Society Clinical Practice Guideline. J Clin Endocrinol Metab. 2017;102:3869–903. 28945902 10.1210/jc.2017-01658

[4] Ross MB, van de Grift TC, Elaut E, Nieder TO, Becker-Hebly I, Heylens G, et al. Experienced barriers of care within European treatment seeking transgender individuals: a multicenter ENIGI follow-up study. Int J Transgender Health. 2023. 10.1080/26895269.2021.1964409. 10.1080/26895269.2021.1964409 PMC9879197 36713146

[5] Burchell D, Coleman T, Travers R, Aversa I, Schmid E, Coulombe S, et al. ‘I don’t want to have to teach every medical provider’: barriers to care among non-binary people in the Canadian healthcare system. Cult Health Sex. 2023. 10.1080/13691058.2023.2185685. 37173293 10.1080/13691058.2023.2185685

[6] Mills TJ, Riddell KE, Price E, Smith DRR. ‘Stuck in the system’: an interpretative phenomenological analysis of transmasculine experiences of gender transition in the UK. Qual Health Res. 2023. 10.1177/10497323231167779. 37018660 10.1177/10497323231167779

[7] Burgwal A, Motmans J. Trans and gender diverse people’s experiences and evaluations with general and trans-specific healthcare services: a cross-sectional survey. Int J Impot Res. 2021. 10.1038/s41443-021-00432-9. 10.1038/s41443-021-00432-9 33854204

[8] Heng A, Heal C, Banks J, Preston R. 2018;Transgender peoples’ experiences and perspectives about general healthcare: a systematic review. Int J Transgenderism. 2018. 10.1080/15532739.2018.1502711.

[9] Van Borm H, Dhoop M, Van Acker A, Baert S. What does someone’s gender identity signal to employers? Int J Manpow. 2020;41:753–77. 10.1108/IJM-03-2019-0164.

[10] Van Borm H, Baert S. What drives hiring discrimination against transgenders? Int J Manpow. 2018. 10.1108/IJM-09-2017-0233.

[11] Vennix P. Transgenders and work: a study of the employment situation of transgenders in the Netherlands and Flanders (Transgenders en werk: een onderzoek naar de arbeidssituatie van transgenders in Nederland en Vlaanderen). Utrecht: Rutgers Nisso Groep; 2010.

[12] Gut T, Arevshatian L, Beauregard TA. HRM and the case of transgender workers: a complex landscape of limited HRM “know how” with some pockets of good practice. Hum Resour Manag Int Dig. 2018. 10.1108/HRMID-06-2017-0121.

[13] Budge SL, Tebbe EN, Howard KAS. The work experiences of transgender individuals: negotiating the transition and career decision-making processes. J Couns Psychol. 2010;57:377–97. 10.1037/a0020472.

[14] Brewster ME, Mennicke A, Velez BL, Tebbe E. Voices from beyond: a thematic content analysis of transgender employees’ workplace experiences. Psychol Sex Orientat Gend Divers. 2014. 10.1037/sgd0000030.

[15] Ozturk MB, Tatli A. Gender identity inclusion in the workplace: broadening diversity management research and practice through the case of transgender employees in the UK. Int J Hum Resour Manag. 2016;27:781–802. 10.1080/09585192.2015.1042902.

[16] Goryunova E, Schwartz AK, Turesky EF. Exploring workplace experiences of transgender individuals in the USA. Gend Manag Int J. 2022. 10.1108/GM-02-2020-0055.

[17] Arijs Q, Burgwal A, Van Wiele J, Motmans J. The price to pay for being yourself: experiences of microaggressions among non-binary and genderqueer (NBGQ) youth. Healthcare. 2023. 10.3390/healthcare11050742. 36900746 10.3390/healthcare11050742 PMC10000855

[18] Dray KK, Smith VRE, Kostecki TP, Sabat IE, Thomson CR. Moving beyond the gender binary: examining workplace perceptions of nonbinary and transgender employees. Gend Work Organ. 2020;27:1181–91. 10.1111/gwao.12455.

[19] Davidson S. Gender inequality: nonbinary transgender people in the workplace. Cogent Soc Sci. 2016. 10.1080/23311886.2016.1236511.

[20] Jeanes E, Janes K. Trans men doing gender at work. Gend Work Organ. 2021. 10.1111/gwao.12675.

[21] Meyer IH. Prejudice, social stress, and mental health in lesbian, gay, and bisexual populations: conceptual issues and research evidence. Psychol Bull. 2003;129:674. 12956539 10.1037/0033-2909.129.5.674 PMC2072932

[22] Bockting WO, Miner MH, Swinburne Romine RE, Hamilton A, Coleman E. Stigma, mental health, and resilience in an online sample of the US transgender population. Am J Public Health. 2013;103:943–51. 10.2105/AJPH.2013.301241. 23488522 10.2105/AJPH.2013.301241 PMC3698807

[23] Cancelliere C, Donovan J, Stochkendahl MJ, Biscardi M, Ammendolia C, Myburgh C, et al. Factors affecting return to work after injury or illness: best evidence synthesis of systematic reviews. Chiropr Man Ther. 2016;24:32. 10.1186/s12998-016-0113-z. 10.1186/s12998-016-0113-z PMC5015229 27610218

[24] Lederer V, Rivard M, Mechakra-Tahiri SD. Gender differences in personal and work-related determinants of return-to-work following long-term disability: a 5-year cohort study. J Occup Rehabil. 2012. 10.1007/s10926-012-9366-0. 22466435 10.1007/s10926-012-9366-0

[25] De Rijk A, Nijhuis F, Alexanderson K. Gender differences in work modifications and changed job characteristics during the return-to-work process: a prospective cohort study. J Occup Rehabil. 2009. 10.1007/s10926-009-9168-1. 19247817 10.1007/s10926-009-9168-1

[26] Fisker J, Hjorthøj C, Hellström L, Mundy SS, Rosenberg NG, Eplov LF. Predictors of return to work for people on sick leave with common mental disorders: a systematic review and meta-analysis. Int Arch Occup Environ Health. 2022;95:1–13. 10.1007/s00420-021-01827-3. 35106629 10.1007/s00420-021-01827-3

[27] White C, Green RA, Ferguson S, Anderson SL, Howe C, Sun J, et al. The influence of social support and social integration factors on return to work outcomes for individuals with work-related injuries: a systematic review. J Occup Rehabil. 2019. 10.1007/s10926-018-09826-x. 30671774 10.1007/s10926-018-09826-x PMC6675768

[28] Etuknwa A, Daniels K, Eib C. Sustainable return to work: a systematic review focusing on personal and social factors. J Occup Rehabil. 2019. 10.1007/s10926-019-09832-7. 30767151 10.1007/s10926-019-09832-7 PMC6838034

[29] Cullen KL, Irvin E, Collie A, Clay F, Gensby U, Jennings PA, et al. Effectiveness of workplace interventions in return-to-work for musculoskeletal, pain-related and mental health conditions: an update of the evidence and messages for practitioners. J Occup Rehabil. 2018;28:1–15. 10.1007/s10926-016-9690-x. 28224415 10.1007/s10926-016-9690-x PMC5820404

[30] Nazarov S, Manuwald U, Leonardi M, Silvaggi F, Foucaud J, Lamore K, et al. Chronic diseases and employment: which interventions support the maintenance of work and return to work among workers with chronic illnesses? a systematic review. Int J Environ Res Public Health. 2019;16:1864. 10.3390/ijerph16101864. 31137817 10.3390/ijerph16101864 PMC6572561

[31] Nieuwenhuijsen K, Verbeek JH, Neumeyer-Gromen A, Verhoeven AC, Bültmann U, Faber B. Interventions to improve return to work in depressed people. Cochrane Database Syst Rev. 2020. 10.1002/14651858.CD006237.pub4. 33052607 10.1002/14651858.CD006237.pub4 PMC8094165

[32] Engel GL. The need for a new medical model: a challenge for biomedicine. Science. 1977;196:129–36. 10.1126/science.847460. 847460 10.1126/science.847460

[33] Nilsen DA, Nissen O, Nordfjærn T, Hara KW, Stiles TC. Who returns to work? Exploring the role of interpersonal problems in occupational rehabilitation. J Occup Rehabil. 2023. 10.1007/s10926-022-10091-2. 37340280 10.1007/s10926-022-10091-2 PMC10495481

[34] Löfgren M, Rembeck G, Hange D, Björkelund C, Svenningsson I, Nordeman L. Promoting health literacy and sense of coherence in primary care patients with long-term impaired work ability—a pilot study. Scand J Prim Health Care. 2022. 10.1080/02813432.2022.2159191. 36622201 10.1080/02813432.2022.2159191 PMC9848260

[35] Jørgensen MB, Larsen AK. Occupational health literacy: Healthy decisions at work. International Handbook of Health Literacy: research, practice and policy across the lifespan. Bristol: Policy Press; 2019, p. 347–58

[36] Tjulin Å, Maceachen E. The importance of workplace social relations in the return to work process a missing piece in the return to work puzzle? In: Gatchel RJ, Schultz IZ, editors. Handbook of return to work from research to practice boston. Boston: Springer, US; 2016. p. 81–97.

[37] Van de Cauter J, Van Schoorisse H, Van de Velde D, Motmans J, Braeckman L. Return to work of transgender people: a systematic review through the blender of occupational health. PloS one. 2021. 10.1371/journal.pone.0259206. 34723993 10.1371/journal.pone.0259206 PMC8559954

[38] Levesque J-F, Harris MF, Russell G. Patient-centred access to health care: conceptualising access at the interface of health systems and populations. Int J Equity Health. 2013;12:1–9. 23496984 10.1186/1475-9276-12-18 PMC3610159

[39] Dietscher C, Pelikan J, Bobek J, Nowak P. The Action Network on Measuring Population and Organizational Health Literacy (M-POHL): a network under the umbrella of the WHO European Health Information Initiative (EHII); Public health panorama. 2019;5:5–69.

[40] Sørensen K. Defining health literacy: Exploring differences and commonalities. In: Okan O, Bauer U, Levin-Zamir D, Pinheiro P, Sørensen K, editors. International Handbook of Health Literacy. Bristol: Policy Press; 2019. p. 5–20.

[41] Ehmann AT, Ög E, Rieger MA, Siegel A. Work-related health literacy: a scoping review to clarify the concept. Int J Environ Res Public Health. 2021. 10.3390/ijerph18199945. 34639262 10.3390/ijerph18199945 PMC8507793

[42] Soellner R, Lenartz N, Rudinger G. Concept mapping as an approach for expert-guided model building: the example of health literacy. Eval Program Plann. 2017;60:245–53. 10.1016/j.evalprogplan.2016.10.007. 27771012 10.1016/j.evalprogplan.2016.10.007

[43] Sørensen K, Van den Broucke S, Fullam J, Doyle G, Pelikan J, Slonska Z, et al. Health literacy and public health: a systematic review and integration of definitions and models. BMC Public Health. 2012. 10.1186/1471-2458-12-80. 22276600 10.1186/1471-2458-12-80 PMC3292515

[44] Charafeddine R, Demarest S, Berete F. Health Literacy (Littératie en santé). Enq Santé 2018.

[45] Pelikan JM, Ganahl K, Van den Broucke S, Sørensen K. Measuring health literacy in Europe: introducing the European Health Literacy Survey Questionnaire (HLS-EU-Q). In: Okan O, Bauer U, Levin-Zamir D, Pinheiro P, Sørensen K, editors. International Handbook of Health Literacy. Bristol: Policy Press; 2019. p. 115–38.

[46] Huffman AH, Mills MJ, Howes SS, Albritton MD. Workplace support and affirming behaviors: Moving toward a transgender, gender diverse, and non-binary friendly workplace. Int J Transgender Health. 2021;22:225–42. 10.1080/26895269.2020.1861575 PMC8118231 34240067

[47] Valente PK, Dworkin JD, Dolezal C, Singh AA, LeBlanc AJ, Bockting WO. Prospective relationships between stigma, mental health, and resilience in a multi-city cohort of transgender and nonbinary individuals in the United States, 2016–2019. Soc Psychiatry Psychiatr Epidemiol. 2022. 10.1007/s00127-022-02270-6. 35312828 10.1007/s00127-022-02270-6

[48] Oorthuys AO, Ross M, Kreukels BP, Mullender MG, van de Grift TC. Identifying coping strategies used by transgender individuals in response to stressors during and after gender-affirming treatments—an explorative study. Healthcare. 2022;11:89. 36611552 10.3390/healthcare11010089 PMC9818796

[49] Law CL, Martinez LR, Ruggs EN, Hebl MR, Akers E. Trans-Parency in the workplace: how the experiences of transsexual employees can be improved. J Vocat Behav januari. 2011;79:710–23.

[50] Budge SL, Katz-Wise SL, Tebbe EN, Howard KAS, Schneider CL, Rodriguez A. Transgender emotional and coping processes: facilitative and avoidant coping throughout gender transitioning. Couns Psychol. 2013. 10.1177/0011000011432753.

[51] Riggs DW, Coleman K, Due C. Healthcare experiences of gender diverse Australians: a mixed-methods, self-report survey. BMC Public Health. 2014;14:1–5. 24597614 10.1186/1471-2458-14-230 PMC3973980

[52] Federal Public Service Employment, Labor, and Social Dialogue. Codex on well-being at work Book I.- General principles Title 4.- Measures related to health surveillance of workers (Codex over het welzijn op het werk Boek I.- Algemene beginselen Titel 4.– Maatregelen in verband met het gezondheidstoezicht op de werknemers). Brussels: 1996.

[53] Lambrechts M-C, Ketterer F, Symons L, Mairiaux P, Peremans L, Remmen R, et al. The approach taken to substance abuse by occupational physicians. J Occup Environ Med. 2015;57:1228–35. 26539772 10.1097/JOM.0000000000000549

[54] Jain A, Hassard J, Leka S, Di Tecco C, Iavicoli S. The role of occupational health services in psychosocial risk management and the promotion of mental health and well-being at work. Int J Environ Res Public Health. 2021;18:3632. 33807352 10.3390/ijerph18073632 PMC8036601

[55] Verlinden H. Key figures absenteeism in 2020 (Kerncijfers Absenteïsme in 2020). Brussels: Securex Corporate EESV; 2021.

[56] Federal Public Service Employment, Labor, and Social Dialogue. Royal Decree of 11/09/2022 amending the codex on well-being at work with regard to the reintegration process for disabled employees (Koninklijk Besluit van 11/09/2022 tot wijziging van de codex over het welzijn op het werk wat het re-integratietraject voor arbeidsongeschikte werknemers betreft). Brussels: Belgisch Staatsblad; 2022.

[57] European Professional Association for Transgender Health. EPATH research policy. EPATH 2019. https://epath.eu/epath-research-policy/. Accessed September 82023.

[58] Vincent BW. Studying trans: recommendations for ethical recruitment and collaboration with transgender participants in academic research. Psychol Sex. 2018. 10.1080/19419899.2018.1434558.

[59] T’Sjoen G, Motmans J. Integrating transgender care into mainstream medicine—an essay by Guy T’Sjoen and Joz Motmans. BMJ. 2022. 10.1136/bmj.o1949. 36191953 10.1136/bmj.o1949 PMC9527634

[60] Mason J. Six strategies for mixing methods and linking data in social science research 2006.

[61] Mertens DM. Transformative research and evaluation. New York: Guilford press; 2008.

[62] Fetters MD, Curry LA, Creswell JW. Achieving integration in mixed methods designs—principles and practices. Health Serv Res. 2013;48:2134–56. 24279835 10.1111/1475-6773.12117 PMC4097839

[63] Maxwell JA, Loomis DM. Mixed methods design: An alternative approach. Handbook of mixed methods in social and behavioral research, vol. 1. Thousand Oaks, CA: SAGE; 2003, p. 241–272.

[64] Maxwell JA, Chmiel M, Rogers SE. Designing integration in multimethod and mixed methods research. In: Hesse-Biber S, Johnson RB, editors. The Oxford Handbook of Multimethod and Mixed Methods Research Inquiry Oxf. Handb. Multimethod Mixed Methods Research Inquiry. New York: Oxford University Press; 2015. p. 223–9.

[65] Thornberg R. Informed grounded theory. Scand J Educ Res. 2012. 10.1080/00313831.2011.581686.

[66] Creswell John W, Clark Vicki PL. Designing and Conducting Mixed Methods Research. 3rd ed. Thousand Oakes: SAGE Publications Inc.; 2018.

[67] von Elm E, Altman DG, Egger M, Pocock SJ, Gøtzsche PC, Vandenbroucke JP. The Strengthening the Reporting of Observational Studies in Epidemiology (STROBE) statement: guidelines for reporting observational studies. J Clin Epidemiol. 2008. 10.1016/j.jclinepi.2007.11.008. 10.1016/j.jclinepi.2007.11.008 18313558

[68] Tong A, Sainsbury P, Craig J. Consolidated Criteria for Reporting Qualitative Research (COREQ): a 32-item checklist for interviews and focus groups. Int J Qual Health Care. 2007. 10.1093/intqhc/mzm042. 17872937 10.1093/intqhc/mzm042

[69] Levitt HM, Bamberg M, Creswell JW, Frost DM, Josselson R, Suárez-Orozco C. Journal article reporting standards for qualitative primary, qualitative meta-analytic, and mixed methods research in psychology: the APA Publications and Communications Board Task Force Report. Am Psychol. 2018. 10.1037/amp0000151. 29345485 10.1037/amp0000151

[70] Teddlie C, Yu F. Mixed methods sampling: a typology with examples. J Mix Methods Res. 2007. 10.1177/1558689806292430.

[71] Michael Quinn P. Qualitative research & evaluation methods: integrating theory and practice. 4th ed. Thousand Oakes: SAGE Publications Inc.; 2015.

[72] Browne K. Snowball sampling: using social networks to research non-heterosexual women. Int J Soc Res Methodol. 2005. 10.1080/1364557032000081663.

[73] Smiley A, Burgwal A, Orre C, Summanen E, García Nieto I, Vidić J, et al. Overdiagnosed but underserved: trans healthcare in Georgia, Poland, Serbia, Spain, and Sweden: Trans Health Survey 2017.

[74] Harris PA, Taylor R, Thielke R, Payne J, Gonzalez N, Conde JG. Research Electronic Data Capture (REDCap)–a metadata-driven methodology and workflow process for providing translational research informatics support. J Biomed Inform. 2009. 10.1016/j.jbi.2008.08.010. 18929686 10.1016/j.jbi.2008.08.010 PMC2700030

[75] International Standard Classification of Education (ISCED). Eurostat Statistics Explained. https://ec.europa.eu/eurostat/statistics-explained/index.php?title=International_Standard_Classification_of_Education_(ISCED). Accessed June 22 2023.

[76] Eurostat. Education and Training- Methodology. https://ec.europa.eu/eurostat/web/education-and-training/methodology. Accessed June 22 2023.

[77] Ahrendt D, Anderson R, Dubois H, Jungblut J-M, Leončikas T, Sándor E, et al. European Quality of Life Survey 2016. Ireland: Eurofound; 2018.

[78] Clays E, Kittel F, Godin I, De Bacquer D, De Backer G. Measures of work-family conflict predict sickness absence from work. J Occup Environ Med. 2009;51:879–86. 19667836 10.1097/JOM.0b013e3181aa5070

[79] Pelikan JM, Ganahl K, Roethlin F. Health literacy as a determinant, mediator and/or moderator of health: empirical models using the European Health Literacy Survey dataset. Glob Health Promot. 2018. 10.1177/1757975918788300. 30427258 10.1177/1757975918788300

[80] Saldaňa J. The Coding Manual for Qualitative Researchers. 4E ed. London: SAGE Publications Inc; 2021.

[81] Charmaz K. Constructing Grounded Theory. 2nd ed. London: SAGE Publications Inc.; 2014.

[82] Vander Sijpe F, Bosmans G. Employee turnover in 2018 (Personeelsverloop in 2018). Brussel: Securex; 2019.

[83] Koehler A, Motmans J, Mulió Alvarez L, Azul D, Badalyan K, Basar K, et al. How the COVID-19 pandemic affects transgender health care-a cross-sectional online survey in 63 upper-middle-income and high-income countries. Int J Transgender Health. 2021;381:2451. 10.1080/26895269.2021.1986191 PMC10373616 37519919

[84] Burgwal A, Gvianishvili N, Hård V, Kata J, García Nieto I, Orre C, et al. Health disparities between binary and non-binary trans people: a community-driven survey. Int J Transgenderism. 2019. 10.1080/15532739.2019.1629370. 10.1080/15532739.2019.1629370 PMC6831016 32999608

[85] Axfors C, Iliadis SI, Rasmusson LL, Beckman U, Fazekas A, Frisén L, et al. Preferences for gender affirming treatment and associated factors among transgender people in sweden. Sex Res Soc Policy. 2021. 10.1007/s13178-021-00650-2.

[86] Hu AC, Liu MT, Chan CH, Gupta S, Dang BN, Ng GY, et al. Gender affirming surgery in nonbinary patients: a single institutional experience. Arch Plast Surg. 2023;50:063–9. 10.1055/s-0042-1758383 PMC9902078 36755659

[87] Swan J, Phillips TM, Sanders T, Mullens AB, Debattista J, Brömdal A. Mental health and quality of life outcomes of gender-affirming surgery: a systematic literature review. J Gay Lesbian Ment Health. 2023;27:2–45.

[88] Bouman M-B, van der Sluis WB, van Woudenberg Hamstra LE, Buncamper ME, Kreukels BP, Meijerink WJ, et al. Patient-reported esthetic and functional outcomes of primary total laparoscopic intestinal vaginoplasty in transgender women with penoscrotal hypoplasia. J Sex Med. 2016;13:1438–44. 27475240 10.1016/j.jsxm.2016.06.009

[89] van der Sluis WB, Bouman M-B, de Boer NK, Buncamper ME, van Bodegraven AA, Neefjes-Borst EA, et al. Long-term follow-up of transgender women after secondary intestinal vaginoplasty. J Sex Med. 2016;13:702–10. 26928775 10.1016/j.jsxm.2016.01.008

[90] Sørensen K, Pelikan JM, Röthlin F, Ganahl K, Slonska Z, Doyle G, et al. Health literacy in Europe: comparative results of the European Health Literacy Survey (HLS-EU). Eur J Public Health. 2015;25:1053–1058. 10.1093/eurpub/ckv043. 25843827 10.1093/eurpub/ckv043 PMC4668324

[91] Rollin L, Ladner J, Gislard A, Monfrin F, Larchevesque J-Y, Deslandes P, et al. Hazard information needs and information seeking in French workers. Occup Med. 2013;63:473–8. 10.1093/occmed/kqt091 23925975

[92] Hostetter CR, Call J, Gerke DR, Holloway BT, Walls NE, Greenfield JC. “We are doing the absolute most that we can, and no one is listening”: barriers and facilitators to health literacy within transgender and nonbinary communities. Int J Environ Res Public Health. 2022. 10.3390/ijerph19031229. 35162254 10.3390/ijerph19031229 PMC8834767

[93] Ståhl C, Karlsson EA, Sandqvist J, Hensing G, Brouwer S, Friberg E, et al. Social insurance literacy: a scoping review on how to define and measure it. Disabil Rehabil. 2021. 10.1080/09638288.2019.1672111. 31589073 10.1080/09638288.2019.1672111

[94] Storms H, Aertgeerts B, Vandenabeele F, Claes N. General practitioners’ predictions of their own patients’ health literacy: a cross-sectional study in Belgium. BMJ Open. 2019. 10.1136/bmjopen-2019-029357. 31519674 10.1136/bmjopen-2019-029357 PMC6747646

[95] Pößnecker T, Baxendale M, Braun S, Schwarz E, Hölzer M, Angerer P, et al. Occupational physicians dealing with mental health: between employee and company interests: a qualitative study. BMC Psychol. 2022. 10.1186/s40359-022-01012-2. 36517913 10.1186/s40359-022-01012-2 PMC9749363

[96] Los FS, de Boer AG, van der Molen HF, Hulshof CT. The implementation of workers’ health surveillance by occupational physicians: a survey study. J Occup Environ Med. 2019;61:e497-502. 31626069 10.1097/JOM.0000000000001740

[97] Nicholson PJ. Occupational medicine: paradise lost. Occup Med. 2017;67:507–9. 10.1093/occmed/kqx122 29048599

[98] Heikkinen AM, Wickström GJ, Leino-Kilpi H, Katajisto J. Privacy and dual loyalties in occupational health practice. Nurs Ethics. 2007;14:675–90. 17901177 10.1177/0969733007077891

[99] Schaap R, Schaafsma F, Huijsmans M, Bosma A, Boot C, Anema J. A context analysis with stakeholders’ views for future implementation of interventions to prevent health problems among employees with a lower socioeconomic position. J Occup Rehabil. 2021. 10.1007/s10926-021-10010-x. 34731392 10.1007/s10926-021-10010-x PMC8564794

[100] Henderson ER. A comparison of health-related quality of life among transgender adults in the United States. J Homosex. 2022. 10.1080/00918369.2021.1892406. 33724155 10.1080/00918369.2021.1892406

[101] Papadaki V, Ntiken A. “As a trans person you don’t live. You merely try to survive and apologize every day for who you are” -Discrimination experiences among trans individuals in Greece. J Homosex. 2022;70:1325–1347. 10.1080/00918369.2021.2020544. 35007491 10.1080/00918369.2021.2020544

[102] Burgwal A, Gvianishvili N, Hård V, Kata J, Nieto IG, Orre C, et al. The impact of training in transgender care on healthcare providers competence and confidence: a cross-sectional survey. Healthcare. 2021;9:967. 34442104 10.3390/healthcare9080967 PMC8391671

[103] Porter S, Lexén A, Bejerholm U. Mental health literacy among vocational rehabilitation professionals and their perception of employers in the return-to-work process. J Vocat Rehabil. 2019;50:157–69.

