# Supplementary material for: Exploring Work Absences and Return to Work During Social Transition and Following Gender-Affirming Care, a Mixed-Methods Approach: ‘Bridging Support Actors Through Literacy’
Source: J Occup Rehabil. 2023 Oct 21;34(2):425–46. doi: 10.1007/s10926-023-10139-x (PMC11180020; doi:10.1007/s10926-023-10139-x)
Supplement: Supplementary file 1 — Supplementary file1 (PDF 417 KB)—Supplementary information 1 (‘Online Resource 1’) is a term of reference and overview of two health literacy models (Figures 1-3) [file 10926_2023_10139_MOESM1_ESM.pdf]

Supplementary information 1 : Terms of reference and HL models

Exploring work absences and return to work during social transition and following gender-affirming care, a mixed-methods approach: 'bridging support actors through literacy.'

Journal of Occupational Rehabilitation

Joy Van de Cauter[1*], Dominique Van de Velde[2], Joz Motmans[3], Els Clays[4], Lutgart Braeckman[1]

[1]Department of Public Health and Primary Care, Unit of Occupational and Insurance Medicine, Faculty of Medicine and Health Sciences, Ghent University, 10 Corneel Heymanslaan, 9000 Ghent, Belgium
[2]Department of Rehabilitation Sciences, Faculty of Medicine and Health Sciences, Ghent University, 10 Corneel Heymanslaan, Ghent, Belgium
[3]Centre for Sexology and Gender, Ghent University Hospital, 10 Corneel Heymanslaan, 9000 Ghent, Belgium
[4]Department of Public Health and Primary Care, Unit of Epidemiology and Prevention, Faculty of Medicine and Health Sciences, Ghent University, 10 Corneel Heymanslaan, 9000 Ghent, Belgium
*Corresponding author
E-mail address: joy.vandecauter@ugent.be

Gender (identity) definitions

Gender identity refers to a person's deeply felt, internal, intrinsic sense of their own gender. Cisgender refers to people whose current gender identity corresponds to the sex they were assigned at birth [1]. The Standards of Care, version 8 [1], created by the World Professional Organization for Transgender Health (WPATH), recommend using transgender and gender diverse (TGD) people as a broader umbrella term. Transgender or trans people are umbrella terms for those whose gender identity and expressions differ from their sex assigned at birth [1]. The term 'gender diverse' (GD) is used to refer to persons whose gender identity (including their gender expression) is at odds with what is perceived as social and cultural gender norms [1]. This includes those who do not place themselves in the male/female binary, such as non-binary, gender fluid, genderqueer, bigender, and polygender identities.

Expressing gender beyond that of the binary conformity has met stigma in a global fashion, which can lead to discrimination and prejudice resulting in 'minority stress'. The latter is a unique socially induced stressor (in addition to people experiencing general stressors) resulting in mental health disparities and negative physical outcomes of TGD people [1].

Health literacy models and definitions

European Health Literacy Survey (EU-HLS)

*"Health literacy is linked to literacy and entails the motivation, knowledge and competencies to access, understand, appraise and apply health information in order to make judgements and take decisions in everyday life concerning healthcare, disease prevention and health promotion to maintain or improve quality of life throughout the course of life". (*Definition of HL from Sørensen et al. 2012 [43])

The European Health Literacy consortium developed the multidimensional comparative 47 questions European Health Literacy Survey (EU-HLS) from a conceptual model of HL and a Delphi process and has been validated in several EU countries. The EU-HLS focuses on three domains (healthcare, prevention of illnesses, and health promotion) incorporating individual, public, and clinical perspectives along with the dynamic aspect of HL 'and to provide an empirical basis for European, national, and regional health policies' (Sørensen et al. 2015[1]).

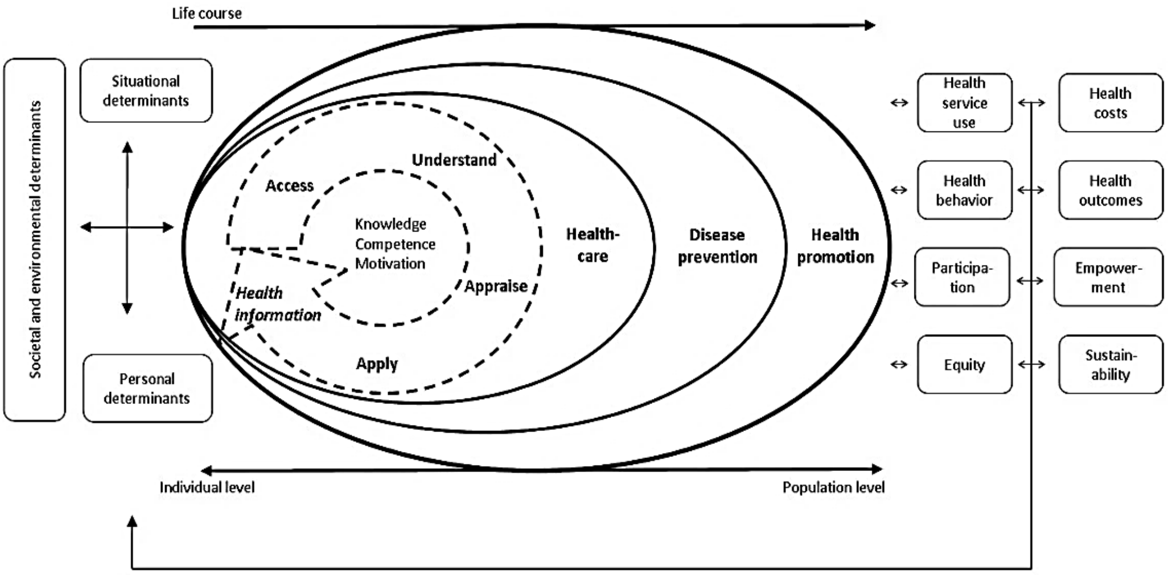

Figure 1 The EU-HLS integrated model of health literacy' (2012; courtesy of Dr. Sørensen and her colleagues under CC by 2.0 license) from [Sørensen, K., Van den Broucke, S., Fullam, J. *et al.* Health literacy and public health: A systematic review and integration of definitions and models. *BMC Public Health* 12, 80 (2012). https://doi.org/10.1186/1471-2458-12-80].

Concept model by Soellner et al.

Soellner's model is a (German-speaking) expert-based concept mapping to develop a HL questionnaire. Perception and behavioral factors play an additional role in this model which consists of nine clusters for HL (self-regulation, self-perception, a proactive approach to health, basic literacy and numeracy skills, information appraisal, information search, health care system knowledge and acting, communication and cooperation, and beneficial personality traits). The structural model (Figure 2-3) frames direct and indirect influences on health (behavior) and the skills for improvement. For further detail on this, the concept map and structural equation model of HL see Soellner, R., Lenartz, N., & Rudinger, G. (2017). Concept mapping as an approach for expert-guided model building: the example of health literacy. *Evaluation and Program Planning*, *60*, 245-253.

[1] Sørensen K, Pelikan JM, Röthlin F, Ganahl K, Slonska Z, Doyle G, Fullam J, Kondilis B, Agrafiotis D, Uiters E, Falcon M, Mensing M, Tchamov K, van den Broucke S, Brand H; HLS-EU Consortium. Health literacy in Europe: comparative results of the European health literacy survey (HLS-EU). Eur J Public Health. 2015 Dec;25(6):1053-8. doi: 10.1093/eurpub/ckv043. Epub 2015 Apr 5. PMID: 25843827; PMCID: PMC4668324.

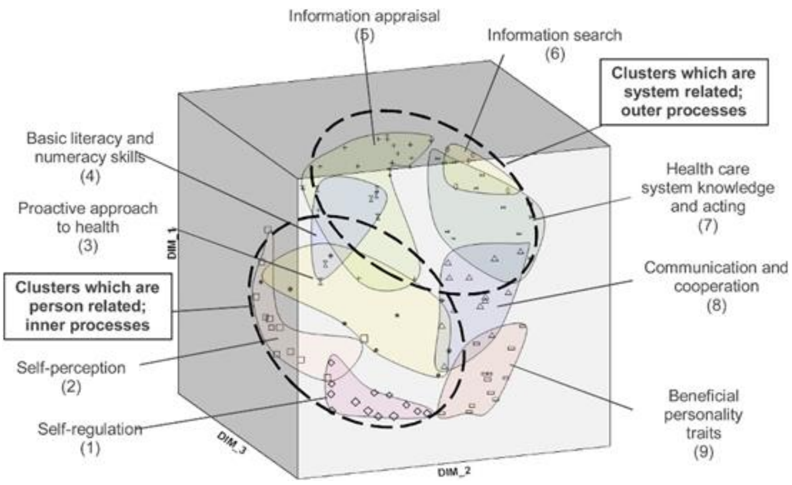

Figure 2 ‘Three-dimensional concept map with nine clusters and illustrating thematic regions of the map.’ from Soellner, R., Lenartz, N., & Rudinger, G. (2017)[42]. Concept mapping as an approach for expert-guided model building: the example of health literacy. Evaluation and Program Planning, 60, 245-253. A license was obtained for print and online reuse (n° 5626530042398).

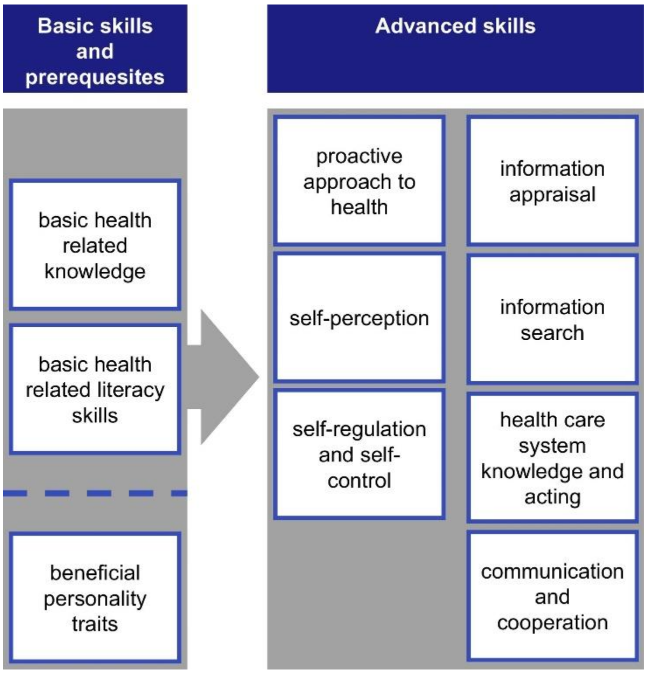

Figure 3 ‘Structural model of health literacy’ by Soellner et al. 2017 from Soellner, R., Lenartz, N., & Rudinger, G. (2017)[42]. Concept mapping as an approach for expert-guided model building: the example of health literacy. Evaluation and Program Planning, 60, 245-253. A license was obtained for print and online reuse (n° 5626530042398)
