# Supplementary material for: Exploring Work Absences and Return to Work During Social Transition and Following Gender-Affirming Care, a Mixed-Methods Approach: ‘Bridging Support Actors Through Literacy’
Source: J Occup Rehabil. 2023 Oct 21;34(2):425–46. doi: 10.1007/s10926-023-10139-x (PMC11180020; doi:10.1007/s10926-023-10139-x)
Supplement: Supplementary file 2 — Supplementary file2 (PDF 216 KB)—Supplementary information 2 (‘Online Resource 2’) contains examples from the questionnaire and interview guide. [file 10926_2023_10139_MOESM2_ESM.pdf]

Supplemental information 2 : Questionnaire and interview guide examples

Exploring work absences and return to work during social transition and following gender-affirming care, a mixed-methods approach: 'bridging support actors through literacy.'

Journal of Occupational Rehabilitation

Joy Van de Cauter[1*], Dominique Van de Velde[2], Joz Motmans[3], Els Clays[4], Lutgart Braeckman[1]

[1]Department of Public Health and Primary Care, Unit of Occupational and Insurance Medicine, Faculty of Medicine and Health Sciences, Ghent University, 10 Corneel Heymanslaan, 9000 Ghent, Belgium

[2]Department of Rehabilitation Sciences, Faculty of Medicine and Health Sciences, Ghent University, 10 Corneel Heymanslaan, Ghent, Belgium
[3]Centre for Sexology and Gender, Ghent University Hospital, 10 Corneel Heymanslaan, 9000 Ghent, Belgium

[4]Department of Public Health and Primary Care, Unit of Epidemiology and Prevention, Faculty of Medicine and Health Sciences, Ghent University, 10 Corneel Heymanslaan, 9000 Ghent, Belgium

*Corresponding author
E-mail address: joy.vandecauter@ugent.be

Examples from the survey questionnaire

We realize that these are personal and sometimes sensitive questions, but we hope you can answer them honestly. All answers serve to better understand and frame the work situation and return to work (= the aim of our research).

1. How would you describe your gender identity at the moment[1]? (multiple answers possible)

- ☐ Man
- ☐ Woman
- ☐ Man with a transgender past
- ☐ Woman with a transgender past
- ☐ A cross-dressing man
- ☐ A cross-dressing woman
- ☐ A trans man: a person who was born female but has a male gender identity
- ☐ A trans woman: a person who was born male but has a female gender identity.
- ☐ Genderqueer
- ☐ Non-binair
- ☐ Polygender
- ☐ Genderfluid
- ☐ Other (complete):. .

---

[1] Based on Motmans J. et al. 2017. Being Transgender in Belgium: 10 years later.(D/2018/10.043/12) [2]

2. We can only use a limited number of categories for analysing the study data. That is why we would like to ask you which group best matches your current gender identity as you define it? Check the answer that fits you best at present.
   I prefer to be in the group of:
   - ☐ (trans) man
   - ☐ (trans) woman
   - ☐ Cross-dresser
   - ☐ Genderqueer / agender / polygender / gender-fluid / non-binary
   - ☐ I don't know, I don't have a preference

3. (If sex registered at birth = F)
   Can you tell us what type of trans-specific healthcare[2] you have already undergone **in the last 5 years**? Please remember that trans-specific healthcare means everything related to your social or medical transition [3]

   Were you incapacitated for work during these steps[4]? If so how long? *Please check the box and fill in*.
   a. I was incapacitated for work after this step or treatment
   b. I resumed my job at the same employer
   c. I resumed work at another employer

If you have not taken any steps, go to the next question XX

	a	b	c	# days of work incapacity
Sociale outing (name, pronouns,..)				
Counseling, gender team support or assessment and/or monitoring by a mental health professional (psychologist / psychiatrist)				
Hormone blockers or puberty blockers				
Gender-affirming hormone treatment (such as testosterone)				
Chest surgery: reducing or removing breasts (mastectomy)				
Removal of uterus / ovaries (hysterectomy / ovariectomy)				
Genital surgery (metoidioplasty, phalloplasty)				
Voice surgery				
Other gender-related surgery				

[2] Based in part on TGEU survey [73]

Example questions of interview guide

“How was your first day of going back to work? “

“What adjustments in the workplace do you think might be necessary? What adjustments would optimize your work environment and tasks?”

“Were there changes in social interactions at work? (e.g. lunch, coffee chat, afterwork)”

“Do you experience that you are assigned a gender role in the workplace by third parties and what do you think about this?”

“What can medical/psychological prevention services (related to the company/work) improve to optimally support future transgender employees in the workplace?”
