# Supplementary material for: Exploring Work Absences and Return to Work During Social Transition and Following Gender-Affirming Care, a Mixed-Methods Approach: ‘Bridging Support Actors Through Literacy’
Source: J Occup Rehabil. 2023 Oct 21;34(2):425–46. doi: 10.1007/s10926-023-10139-x (PMC11180020; doi:10.1007/s10926-023-10139-x)
Supplement: Supplementary file 3 — Supplementary file3 (PDF 304 KB)—Supplementary Information 2 (‘Online Resource 3) contains detailed health literacy and support at work results (Figure 4-5, Table 5). [file 10926_2023_10139_MOESM3_ESM.pdf]

Supplementary information 3: European Health Literacy Survey (HLS-Q6) and support at work

Exploring work absences and return to work during social transition and following gender-affirming care, a mixed-methods approach: 'bridging support actors through literacy.'

Journal of Occupational Rehabilitation

Joy Van de Cauter[1*], Dominique Van de Velde[2], Joz Motmans[3], Els Clays[4], Lutgart Braeckman[1]

[1]Department of Public Health and Primary Care, Unit of Occupational and Insurance Medicine, Faculty of Medicine and Health Sciences, Ghent University, 10 Corneel Heymanslaan, 9000 Ghent, Belgium

[2]Department of Rehabilitation Sciences, Faculty of Medicine and Health Sciences, Ghent University, 10 Corneel Heymanslaan, Ghent, Belgium

[3]Centre for Sexology and Gender, Ghent University Hospital, 10 Corneel Heymanslaan, 9000 Ghent, Belgium

[4]Department of Public Health and Primary Care, Unit of Epidemiology and Prevention, Faculty of Medicine and Health Sciences, Ghent University, 10 Corneel Heymanslaan, 9000 Ghent, Belgium

*Corresponding author
E-mail address: joy.vandecauter@ugent.be

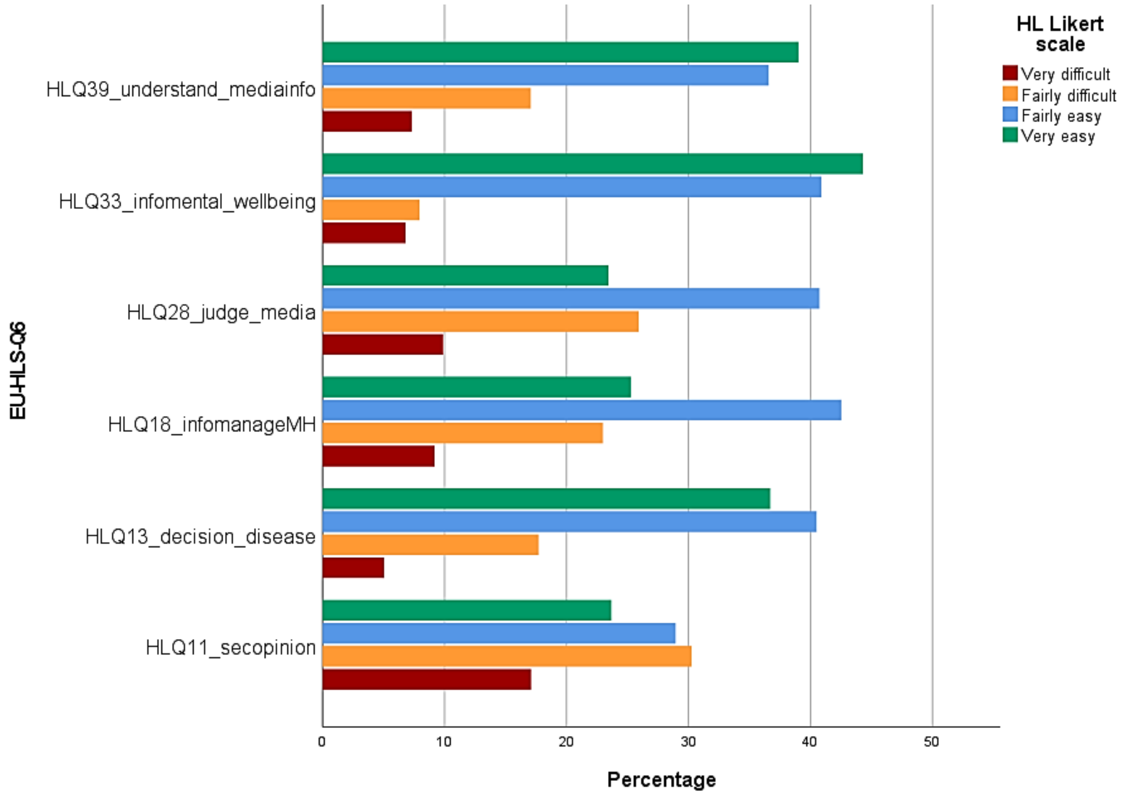

Figure 4: Detailed results of the short form EU-HLS-Q6 of Belgian TGD people

HL score was significantly correlated with health perception (**τ**$_b$=-0195; p=0.03), support from the occupational physician (**τ**$_b$=0.265; p=0.018), support from the internal prevention advisor (**τ**$_b$=0.286, p= 0.040) and support from HR (**τ**$_b$=0.265,p=0.018). Health literacy level was only associated with corporate seniority (χ= 10.96; p=0.027).

Support at work :accompagnying text with figure 5 and table 5

Perceived support at work was significantly higher (Fisher 5.468; p<0.05) when TGD people had a higher corporate seniority ; while age (p=0.658; F 0.605; education (p=1.00), full-timers versus part-timers (p=0.336), hierarchical position at work (p=0.565) or total occupational seniority (p=0.103), working as a supervisor (p=0.684), revealed no significant proportional differences of support.

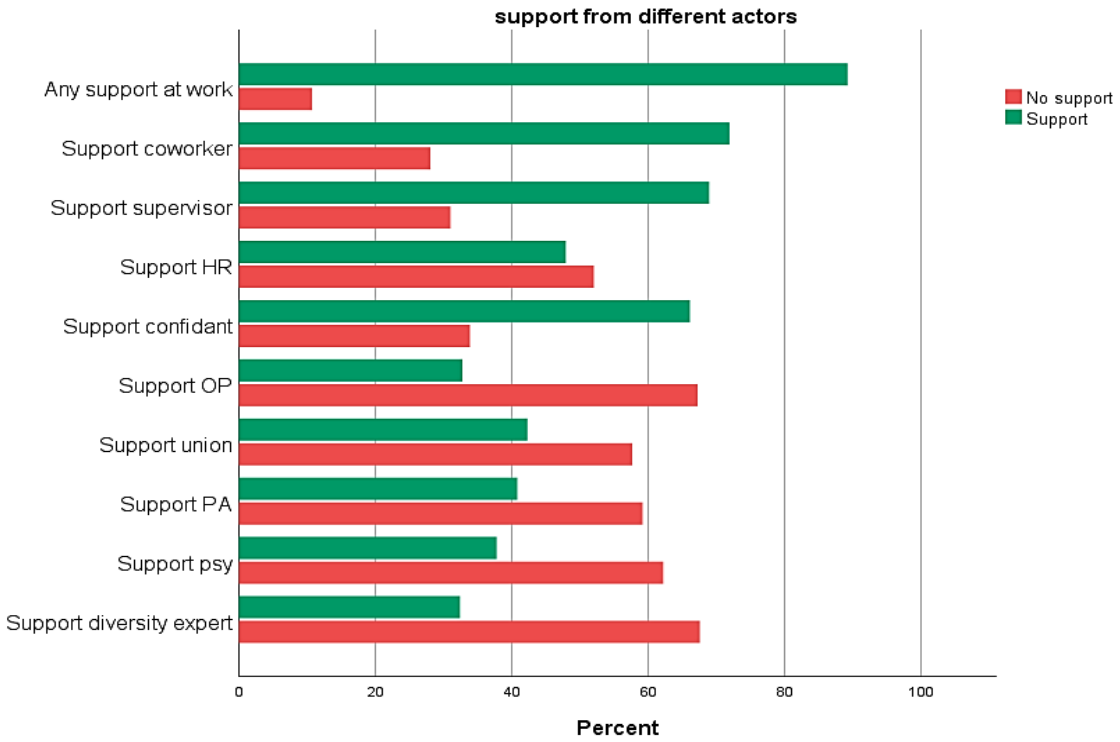

Figure 5: Levels of support of persons and services at work during transition

Table 5: Support at work for total sample and stratified by gender identity

	Total sample (n=128)		Trans men (n=42)		Trans women (n=51)		GD people (n=32)		p
	n	C%	n	R%	n	R%	n	R%	
Any perceived support (n=93)									0.09
no support	10	10.8	3	30.0%	2	20.0%	5	50.0%	
support	83	89.2	33	39.8%	34	41.0%	16	19.3%	
Support Supervisor (n=87)									0.16
no support	27	31.0	13	48.1%	6	22.2%	8	29.6%	
support	60	69.0	22	36.7%	26	43.3%	12	20.0%	
Support coworkers (n=89)									0.27
no support	25	28.1	10	40.0%	7	28.0%	8	32.0%	
support	64	71.9	24	37.5%	28	43.8%	12	18.8%	
Support HR (n=73)									**0.04***
no support	38	52.1	19	50.0%	12	31.6%	7	18.4%	
support	35	47.9	8	22.9%	20	57.1%	7	20.0%	
Support occup physician (n=58)									**<0.01***
no support	39	67.2	18	46.2%	12	30.8%	9	23.1%	
support	19	32.8	2	10.5%	14	73.7%	3	15.8%	
Support internal PA (n=49)									0.38
no support	29	59.2	11	37.9%	11	37.9%	7	24.1%	
support	20	40.8	7	35.0%	11	55.0%	2	10.0%	
Support psychosocial PA (n=45)									0.13
no support	28	62.2	12	42.9%	8	28.6%	8	28.6%	
support	17	37.8	5	29.4%	10	58.8%	2	11.8%	
Support confidant (n=62)									0.13
no support	21	33.9	6	28.6%	8	38.1%	7	33.3%	
support	41	66.1	19	46.3%	17	41.5%	5	12.2%	
Support union (n=52)									0.23
no support	30	57.7	13	43.3%	9	30.0%	8	26.7%	
support	22	42.3	7	31.8%	12	54.5%	3	13.6%	
Support diversity team (n=37)									0.56
no support	25	67.6	11	44.0%	8	32.0%	6	24.0%	
support	12	32.4	3	25.0%	5	41.7%	4	33.3%	

C% column percentage; R%: row percentages; services or persons not applicable were coded as missing for total support; PA: prevention advisor
